# Non-cross-reactive epitopes dominate the humoral immune response to COVID-19 vaccination – kinetics of plasma antibodies, plasmablasts and memory B cells

**DOI:** 10.3389/fimmu.2024.1382911

**Published:** 2024-05-14

**Authors:** Kilian A. Wietschel, Kevin Fechtner, Elmer Antileo, Goran Abdurrahman, Chiara A. Drechsler, Michelle Kudzayi Makuvise, Ruben Rose, Mathias Voß, Andi Krumbholz, Stephan Michalik, Stefan Weiss, Lena Ulm, Philipp Franikowski, Helmut Fickenscher, Barbara M. Bröker, Dina Raafat, Silva Holtfreter

**Affiliations:** ^1^ Institute of Immunology, University Medicine Greifswald, Greifswald, Germany; ^2^ Institute for Infection Medicine, Kiel University and University Medical Center Schleswig-Holstein, Kiel, Germany; ^3^ Labor Dr. Krause und Kollegen MVZ GmbH, Kiel, Germany; ^4^ Interfaculty Institute for Genetics and Functional Genomics, Department of Functional Genomics, University Medicine Greifswald, Greifswald, Germany; ^5^ Friedrich Loeffler-Institute of Medical Microbiology, University Medicine Greifswald, Greifswald, Germany; ^6^ Institute for Educational Quality Improvement, Humboldt University of Berlin, Berlin, Germany; ^7^ Department of Microbiology and Immunology, Faculty of Pharmacy, Alexandria University, Alexandria, Egypt

**Keywords:** COVID-19 vaccination, dynamics, cross-reactive antibodies, plasmablast, memory B cell, HCoV, influenza, original antigenic sin

## Abstract

**Introduction:**

COVID-19 vaccines are highly effective in inducing protective immunity. While the serum antibody response to COVID-19 vaccination has been studied in depth, our knowledge of the underlying plasmablast and memory B cell (Bmem) responses is still incomplete. Here, we determined the antibody and B cell response to COVID-19 vaccination in a naïve population and contrasted it with the response to a single influenza vaccination in a primed cohort. In addition, we analyzed the antibody and B cell responses against the four endemic human coronaviruses (HCoVs).

**Methods:**

Measurement of specific plasma IgG antibodies was combined with functional analyses of antibody-secreting plasmablasts and Bmems. SARS-CoV-2- and HCoV-specific IgG antibodies were quantified with an in-house bead-based multiplexed immunoassay.

**Results:**

The antibody and B cell responses to COVID-19 vaccination reflected the kinetics of a prime-boost immunization, characterized by a slow and moderate primary response and a faster and stronger secondary response. In contrast, the influenza vaccinees possessed robust immune memory for the vaccine antigens prior to vaccination, and the recall vaccination moderately boosted antibody production and Bmem responses. Antibody levels and Bmem responses waned several months after the 2^nd^ COVID-19 vaccination, but were restored upon the 3^rd^ vaccination. The COVID-19 vaccine-induced antibodies mainly targeted novel, non-cross-reactive S1 epitopes of the viral spike protein, while cross-reactive S2 epitopes were less immunogenic. Booster vaccination not only strongly enhanced neutralizing antibodies against an original SARS-CoV-2 strain, but also induced neutralizing antibodies against the Omicron BA.2 variant. We observed a 100% plasma antibody prevalence against the S1 subunits of HCoVs, which was not affected by vaccination.

**Discussion:**

Overall, by complementing classical serology with a functional evaluation of plasmablasts and memory B cells we provide new insights into the specificity of COVID-19 vaccine-induced antibody and B cell responses.

## 1 Introduction

The coronavirus disease 2019 (COVID-19), caused by the severe acute respiratory syndrome coronavirus 2 (SARS-CoV-2), has had an enormous impact on health worldwide, and is still posing a challenge due to the spread of novel variants of concern (VOCs). Vaccination was key in combating the COVID-19 pandemic. In a global effort, COVID-19 vaccines were developed and made widely available with unprecedented speed (1). The mRNA vaccines from Pfizer-BioNTech (Comirnaty^®^, BNT162b2, short: BNT) and Moderna (Spikevax^®^, mRNA-1278, MOD) were licensed in December 2020 and January 2021, respectively. The adenovirus-vectored vaccine from AstraZeneca (Vaxzevria^®^, ChAdOx1-S, AZD) followed at the end of January 2021 (2, 3). All these vaccines are based on the SARS-CoV-2 spike (S) protein, which is essential for SARS-CoV-2 target recognition, adhesion, and host cell entry and triggers a strong neutralizing antibody response. The underlying sequence is derived from the original 2019 SARS-CoV-2 virus. SARS-CoV-2 vaccines have been shown to be safe and highly effective in reducing the severity of symptomatic SARS-CoV-2 infections, as well as preventing hospitalizations and COVID-19-associated deaths (4, 5). As of August 2023, more than 82.5% of the EU adult population has received at least 2 COVID-19 vaccines (1).

Vaccination induces the activation of naïve antigen-specific B cells, which proliferate and differentiate into antibody-secreting plasmablasts, long-lived plasma cells and memory B cells (Bmem). Plasmablasts are short-lived cells responsible for the acute antibody production. A transient plasmablast peak can be detected in the circulation a few days after each vaccine dose, with the second and third doses eliciting earlier and more intense plasmablast bursts (6–9). Plasmablast-derived antibodies provide early protection against the vaccine target. Humoral immune memory, however, is sustained by long-lived plasma cells and Bmems (6, 10). Long-lived plasma cells reside in the bone marrow and provide long-lasting protection against (re-) infection by continuously producing antibodies. In addition, circulating Bmems respond rapidly to a re-encounter with the vaccine or pathogen. They proliferate and differentiate into antibody-secreting plasma cells (11, 12).

Numerous studies have reported that COVID-19 vaccination induces a strong anti-S IgG antibody response (8, 9, 13). These antibodies play a crucial role in neutralizing the virus and mitigating infection (14). However, the antibody levels wane within months of vaccination (8, 15–18), necessitating repeated vaccinations to boost protective antibody levels (19). Recent data illustrate that S-specific Bmems also emerge after the first vaccination. Bmem frequencies rapidly decline after the 2^nd^ vaccination and then stabilize at a lower level (16, 20). A third vaccination restores and enhances the Bmem response (16). While the serum antibody response to COVID-19 vaccination has been studied in depth, our knowledge of the underlying instantaneous B cell response and the B cell memory is still incomplete. Published studies mostly quantify the S-specific plasmablast- and Bmem populations (7–9, 21), but do not assess their functionality.

More recently, the SARS-CoV-2 pandemic was fueled by the emergence of SARS-CoV-2 VOCs, which escape immune recognition and display higher transmissibility (22, 23). Notably, booster vaccinations promote the emergence of broadly reactive antibodies with neutralizing capacity against VOCs (24). Another important aspect of COVID-19 immunity is cross-reactivity with the four endemic human coronaviruses (HCoVs) (21, 25–27). These viruses – HCoV-229E, -NL63, -OC43, and -HKU1 – typically cause 5% of seasonal common colds, usually with mild to moderate respiratory illness (28). Cross-reactive antibodies mainly target the evolutionarily more conserved S2 subunit of the S protein rather than the more variable receptor-binding S1 subunit. Similarly, a considerable proportion of healthy individuals harbors cross-reactive CD4^+^ T cells recognizing conserved epitopes, mostly within the S2 subunit (29, 30). Whether this cross-reactivity is mirrored on plasmablast and Bmem levels remains to be clarified.

Here, we compared the B cell and antibody response to vaccination in two contrasting scenarios: Three consecutive COVID-19 vaccinations in a naïve population versus a single influenza vaccination in a previously exposed cohort. In addition, we examined the pre-existing humoral immunity against endemic HCoVs and its effects on the vaccine response. We combined the measurement of specific plasma antibodies with the functional analysis of antibody-secreting plasmablasts and Bmems. The COVID-19 immunization showed the kinetics of a prime-boost immunization, whereas the influenza vaccination induced a memory response. All study participants had antibodies against all endemic HCoVs at baseline. These pre-existing antibodies cross-reacted moderately with the nucleocapsid protein (NP) and the S2 subunit, but not with the S1 subunit and the receptor-binding domain (RBD), which are less conserved among human coronaviruses. Vaccine-induced antibodies, in contrast, mainly targeted the novel, non-cross-reactive S1 epitopes.

## 2 Materials and methods

### 2.1 Study design and participants

**Figure 1:**
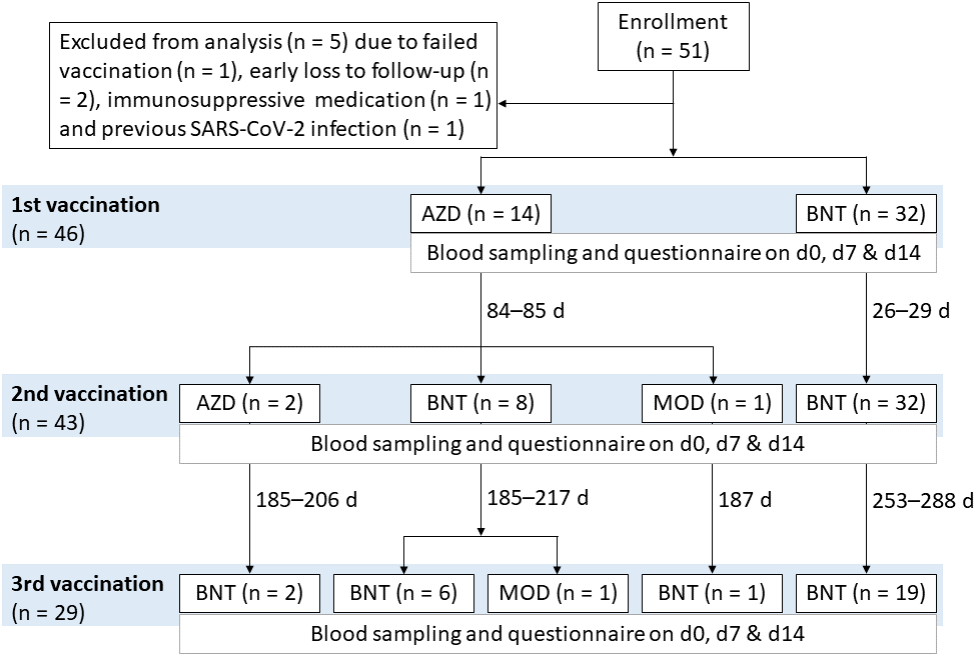
Vaccination regimens of the COVID-19 vaccination study AICOVI. A total of 51 healthy hospital staff of the University Medicine Greifswald were recruited for the AICOVI study. Four subjects were excluded due to failed first vaccination (n = 1), early loss to follow-up after the first blood donation (n = 2) or use of immunosuppressive medication (n = 1). One further subject was excluded from data analyses due to previous SARS-CoV-2 infection. The included subjects (n = 46) received either homologous or heterologous regimens of COVID-19 vaccines: In detail, subjects received either ChAdOx1-S (AZD; n = 14) or BNT162b2 (BNT; n = 32) as first vaccine. Three subjects received only one dose of AZD (incomplete regimen); all others (n = 43) received two immunizations with either (i) two doses of AZD (n = 2; 85 days apart), (ii) one dose each of AZD and BNT (n = 8; 85 days apart), (iii) one dose each of AZD and Spikevax^®^ (MOD; n = 1; 84 days apart), or (iv) two doses of BNT (n = 32; 26–29 days apart), respectively. Additionally, n = 29 received a 3^rd^ vaccination with either BNT (n = 28; 185–288 days after the second vaccination) or MOD (n = 1; 217 days after the 2^nd^ vaccination). Abbreviations: AZD, ChAdOx1-S (Vaxzevria^®^; AstraZeneca); BNT, BNT162b2 (Comirnaty^®^; BioNTech/Pfizer); MOD, Spikevax^®^ (Moderna).

Two exploratory observational longitudinal cohort studies were conducted at the University Medicine Greifswald (UMG, Germany), namely the Adaptive Immune Response after COVID-19 Vaccination (AICOVI study; ClinicalTrial.gov identifier: NCT04826770) and the Adaptive Immune Response after Influenza Vaccination (AIGI study; NCT05129436). The studies aimed to investigate the kinetics of vaccine-specific antibody production and B cell response after SARS-CoV-2 and influenza vaccination, respectively. Inclusion criteria for both studies included: (i) planned vaccination against SARS-CoV-2/influenza; (ii) age ≥ 18 years; (iii) written informed consent; (iv) body mass index ≥ 18.5 kg m^-^²; (v) absence of infectious diseases, blood coagulation disorders, anemia or similar conditions; and (vi) no known congenital or acquired immunodeficiency. For the AICOVI study we recruited a total of 51 healthy healthcare workers of the UMG between 19 and 61 years of age, who had planned to receive their first vaccination against SARS-CoV-2 in January and February 2021. Four subjects were excluded due to failed first vaccination (n = 1), early loss to follow-up after the first blood donation (n = 2), or use of immunosuppressive medication (n = 1). Moreover, one subject was excluded from data analyses due to a previous SARS-CoV-2 infection. Data from the remaining 46 participants were included in the analyses (**Figure 1**; **Supplementary Table 1**; **Supplementary Figure 1**). These 46 probands were considered SARS-CoV-2 naïve based on questionnaire data. SARS-CoV-2 exposure was assessed at each sampling occasion (no positive SARS-CoV-2 test, no respiratory symptoms, or respiratory symptoms but no positive SARS-CoV-2 test). The AIGI study included a total of 17 healthy subjects between 22 and 66 years of age who had planned to receive their influenza vaccination with VaxigripTetra^®^ (Sanofi-Aventis, Lyon, France), a quadrivalent split-virion, inactivated influenza vaccine, in October 2020 (**Supplementary Table 1**). 15 out of 17 subjects were vaccinated with VaxigripTetra^®^ at least once in the last three seasons (2017-2019).

In both studies peripheral blood samples were collected by peripheral venipuncture, preferably from the cubital fossa, on the day of vaccination (d0; AICOVI: up to three vaccinations, AIGI: one vaccination) as well as 7 and 14 days after each vaccination (d7 and d14, respectively). Serum samples were obtained from AIGI subjects, plasma samples from AICOVI subjects. Additionally, plasma samples collected in 2018 from 20 healthy subjects between 25 and 57 years of age (pre-COVID-19 controls, TRP study), and 10 sera from hospitalized convalescent COVID-19 patients who had agreed to donate their blood for a local biobank study (viP) were used for validating the Corona Array (see below).

Participants were also asked to complete standardized questionnaires on each day of blood sampling to gather biometric data including sex, age, weight, and height; as well as data regarding the SARS-CoV-2 vaccination (AICOVI), previous SARS-CoV-2 infections (AICOVI), previous influenza vaccinations (AIGI), other current infections, medication, immune system disorders and secondary vaccination effects. No serious adverse events were reported in the AICOVI and AIGI studies.

### 2.2 Ethics and data protection

The AICOVI, AIGI, TRP and viP studies were approved by the Ethics Committee of the University Medicine Greifswald (internal registration numbers BB 001/21f, 185/20, 043/17a, and 060/20). All work was conducted in accordance with the tenets of the Declaration of Helsinki (version 2013, Fortaleza). All requirements of data protection and confidentiality according to local regulations, the State Data Protection Act Mecklenburg-Western Pomerania, the European Data Protection Directive 95/46/EC, and the General Data Protection Regulation (GDPR) were fully met.

### 2.3 PBMC isolation

Peripheral blood mononuclear cells (PBMCs) were isolated from whole blood using either standard Pancoll density gradient centrifugation (anticoagulant: EDTA, Pancoll 1.077 g/mL; PanBiotech, Aidenbach, Germany; AIGI and AICOVI) or using Vacutainer^®^ cell processing tubes (CPT™ Cell Preparation Tubes with Sodium Heparin; BD, Heidelberg, Germany; AICOVI) according to the manufacturer’s instructions. Isolated PBMCs were resuspended in leukocyte medium (RPMI 1640; PanBiotech) and counted cytometrically using Trucount beads (Trucount Absolute Counting Tubes; BD). PBMCs were diluted to 5 × 10^6^ cells/mL in freezing medium (RPMI medium supplemented with 40% fetal calf serum (FCS; Sigma-Aldrich, Taufkirchen, Germany) and 10% dimethyl sulfoxide (AppliChem, Darmstadt, Germany), aliquoted in cryovials and stored at -156°C for at least six days.

### 2.4 MENSA collection

To obtain medium enriched for newly synthesized antibodies (MENSA) for the analysis of *ex vivo* antibody production, the isolated PBMCs were cultured in IMDM cell culture medium (IMDM supplemented with 10% FCS, 100 U/mL penicillin G sodium, 100 µg/mL streptomycin sulfate, 292 µg/mL L-glutamine (Penicillin-Streptomycin-Glutamine Gibco; Life Technologies, Carlsbad, California, USA) and 50 µmol/L β-mercaptoethanol (Sigma-Aldrich)) under non-stimulating and stimulating conditions. In detail, PBMCs were seeded at 2 × 10^6^ cells/mL in 200 µL IMDM cell culture medium into 96-well round-bottom cell culture plates. PBMCs were either left untreated to assess spontaneous antibody secretion (MENSA) or stimulated with 20 ng/mL IL-2 (Gibco, Life Technologies) and 2 µg/mL of the synthetic dual TLR7/8 agonist resiquimod (R848; Sigma-Aldrich) to assess antibody secretion by Bmems (MENSA+) (31). After 7 days of incubation (37°C, 5% CO_2_), PBMCs were harvested (300 × *g*, 5 min) and the culture supernatants were collected, aliquoted and stored at -80°C until assayed.

### 2.5 Corona Array

The Corona Array is an in-house bead-based 10-plex suspension array based on the xMAP^®^ technology (Luminex^®^, Austin, Texas, USA) for the simultaneous analysis of antibodies of different specificities in one sample. The 10-plex included 6 recombinant His-tagged proteins/protein subunits of SARS-CoV-2 and 4 recombinant S1 subunits of HCoVs (**Supplementary Table 2**). The proteins were covalently coupled to MagPlex magnetic microspheres at 100 µg per 1.25 × 10^7^ beads, and the coupling efficiency was determined as previously described in detail (32).

The Corona Array was performed with plasma as well as MENSA and MENSA+ samples as previously described (32). Briefly, different seven-step dilution series were prepared in bead buffer based on the expected range of signals: (i) 1:20–1:312,500 (plasma); (ii) 1:1–1:64 (MENSA); and (iii) 1:1–1:729 (MENSA+). A plasma pool (prepared from plasma samples of all AICOVI donors on day 14 after the second vaccination) was included on each plate for data normalization. Antibody binding was determined on the BioPlex 200 system (Bio-Rad Laboratories GmbH; Feldkirchen, Germany), with bead buffer serving as blank, using the following instrument settings: bead type: MagPlex beads; beads: 100 beads per region; sample timeout: 60 sec; sample volume: 80 μL; gate settings: 7,500–15,000 (BioPlex Manager 5.0; Bio-Rad Laboratories GmbH).

### 2.6 Influenza ELISA

IgG antibodies against the influenza vaccine in serum, MENSA and MENSA+ of AIGI subjects were detected by an indirect enzyme-linked immunosorbent assay (ELISA). ELISA plates (96-well Nunc-Immuno plates MaxiSorp; Thermo Fisher Scientific; Waltham, Massachusetts, USA) were coated overnight with 1:500 diluted influenza vaccine (VaxigripTetra 2020/2021; Sanofis-Aventis; inactivated split vaccine containing the hemagglutinin of A/Guangdong-Maonan/SWL1536/2019 (H1N1)pdm09, A/Hong Kong/2671/2019 (H3N2), B/Washington/02/2019, and B/Phuket/3073/201-like strains grown in embryonated chicken eggs; final hemagglutinin concentration: 0.24 µg/mL). After washing and blocking steps, serial dilutions of the sera (1:500–1:8,192,000) or MENSA samples (1:1–1:243 or 1:10–1:10,240 for samples with high antibody levels) were added. Bound antibodies were detected by incubation with secondary goat anti-human IgG POD (Jackson ImmunoResearch Labs; Cambridge, U.K.; final concentration: 50 ng/mL). Substrate conversion (TMB Substrate Reagent Set; BD OptEIA, BD Biosciences) was quantified at 450 nm with a Tecan Infinite 200 PRO (Tecan, Männedorf, Switzerland).

### 2.7 Cell-based virus neutralization test

To determine the neutralizing capacity of plasma samples against SARS-CoV-2, they were tested in an in-house Vero cell-based virus neutralization test in triplicates in a 96-well plate format under biosafety level 3 conditions, as previously reported (33–37). Briefly, all samples were diluted from 1:10 to 1:1280 in a DMEM-based FCS-free cell culture medium. Either 50 plaque-forming units per well of a type B.1.513 strain (isolated in April 2020 in Germany, pre-VOC) or an Omicron BA.2 strain (isolated in January 2022 in Germany) were used as antigens; both strains are fully sequenced (35, 36, 38). After an incubation of the plasma dilutions with the virus strains at 37°C for one hour, the serum mixture was added to Vero cells (order no. 605372, CLS Cell Lines Service GmbH, Eppelheim, Germany) and incubated for four (B.1.513) or six (BA.2) days in cell culture medium supplemented with FCS to a final concentration of 10%. The different incubation times were chosen because the BA.2 strain takes considerably longer to induce a clearly recognizable cytopathic effect. Cells were then fixed with paraformaldehyde and stained with an aqueous crystal violet methanol solution. Prevention of the development of a cytopathic effect in ≥ two out of three wells of a plasma dilution step defined the neutralizing antibody titer of the respective sample. If the exact neutralizing antibody titer could not be determined, the geometric mean of the two adjacent titers was calculated. The cut-off for neutralizing antibody detection was set at a titer of > 1:10.

The 11 probands for the neutralization assay were chosen based on the following criteria: (1) homogenous vaccination scheme (BNT/BNT/BNT), (2) complete sample set (three vaccinations, each with d0, d7, and d14 samples), (3) no extreme values in the immune response at any time, (4) lack of other peculiarities (e.g. deviations in sampling).

### 2.8 Data visualization and statistical analysis

During the course of the AICOVI study the recommendations and availability of COVID-19 vaccines changed, resulting in different vaccination schemes within the study (**Figure 1**) (3).

Data obtained from the Corona Array and influenza ELISA were analyzed using the xMAPr software (xMAPr 1.2; S. Michalik; https://github.com/stemicha/xMAPr_app). Blank values were corrected for outliers by excluding the top 5% quantile. Initially assuming a saturation curve regression model, the relative antibody concentration of each sample from the curves of the dilution series was estimated by means of sequential multiple regressions (39). The relative IgG concentration was calculated using the signal intensity (median fluorescence intensity, MFI) and the dilution factor according to the following formula:


$$\text{relative}\;\text{IgG}\;\text{concentration}\;[\text{AU}]=\frac{1}{2}{\text{MFI}}_{\text{max}}\times {\text{dilution}\;\text{factor}}_{\frac{1}{2}{\text{MFI}}_{\text{max}}}$$

If curve fitting failed, the missing relative antibody concentration values were imputed based on a global LOESS fit over a single dilution (AICOVI: plasma: 1:12,500; MENSA: 1:2; MENSA+: 1:9; AIGI: MENSA and MENSA+: 1:27). Imputation was performed for 20/4480 plasma samples, 1862/3570 MENSA samples, and 900/3570 MENSA+ samples from AICOVI, as well as 37/51 MENSA samples and 3/51 MENSA+ samples from AIGI. The frequent lack of S1-specific antibodies in MENSA was expected, because S1-specific plasmablasts (MENSA) are only generated upon antigen exposure and their surge in the peripheral blood upon vaccination only lasts few days (9, 40, 41). Finally, a plate-to-plate normalization of the relative IgG concentrations was performed using the plasma pool (prepared as described above).

Statistical tests and data visualization were performed with GraphPad Prism (v8.0.1) and R Statistical Software (v4.0.5) with additional packages (42, 43). For the AICOVI cohort (paired samples with few missing data points) 13 groups were *a priori* selected for comparison based on their biological relevance. Specifically, pairwise comparisons were made between d0 versus d7 and d14, and d7 versus d14 after each vaccination, as well as between d14 versus d0 and d14 between subsequent vaccinations, resulting in a total of 13 pairwise comparisons. Mixed-effects ANOVAs with Geisser-Greenhouse correction were estimated and followed by Sidak’s multiple group comparisons. For the AIGI cohort (paired samples without missing data points) we used the Friedman test with Dunn’s test for multiple comparisons. A two-tailed Mann-Whitney test was employed for comparison of two datasets. Cut-offs for baseline antibody levels in plasma, MENSA and MENSA+ were defined as median AU[v1d0] + (3 × IQR).

## 3 Results

**Figure 2:**
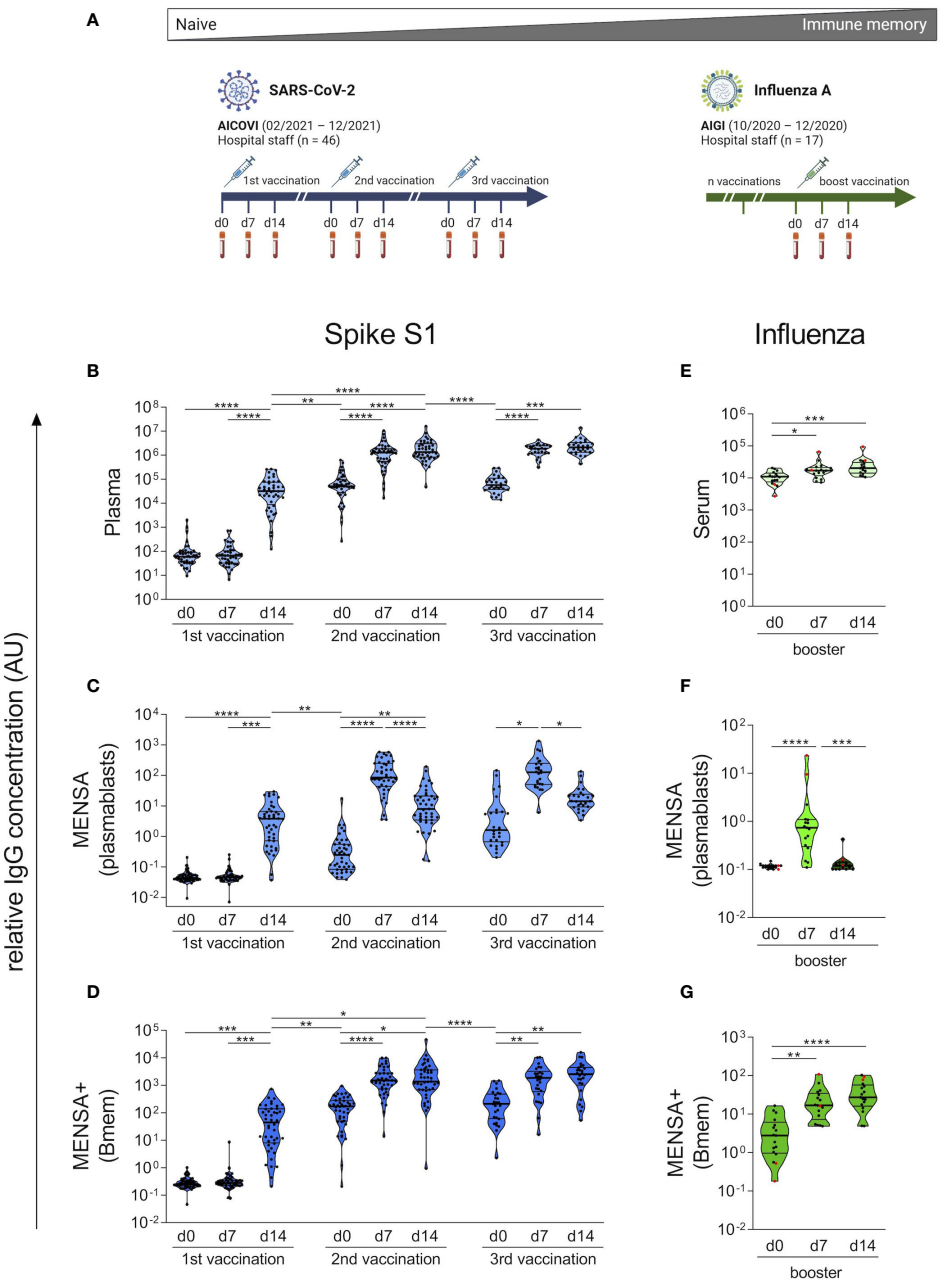
Kinetics of vaccine-induced plasma IgG, plasmablast and Bmem responses strongly differ between COVID-19 and influenza vaccinations. Plasma and peripheral blood mononuclear cells (PBMCs) were obtained from 46 AICOVI subjects on the day of the 1^st^, 2^nd^ and, in a subset of subjects (n = 29), the 3^rd^ COVID-19 vaccination as well as 7 and 14 days later. Similarly, serum and PBMCs were obtained from 17 AIGI subjects on the day of influenza vaccination, as well as 7 and 14 days later **(A)**. PBMCs were cultured and either left untreated to assess spontaneous antibody secretion by plasmablasts (MENSA), or stimulated with IL-2 and the dual TLR7/8 ligand Resiquimod to assess antibody secretion by circulating Bmems (MENSA+). IgG antibodies against the SARS-CoV-2 S1 subunit were quantified in serially diluted plasma **(B)**, MENSA **(C)** and MENSA+ **(D)** using a bead-based multiplexed immunoassay (Corona Array). IgG antibodies against the influenza vaccine VaxiGripTetra^®^ were quantified in serially diluted serum **(E)**, MENSA **(F)** and MENSA+ **(G)** by indirect ELISA. Subjects who did not receive VaxigripTetra^®^ (Sanofi-Aventis) at least once in the 3 past seasons (2017-2019) are shown in red (n = 2). A relative IgG concentration (AU) was calculated by multiplying the half-maximal mean fluorescence intensities with the corresponding dilution factor. Data reflect prime-boost kinetics after COVID-19 vaccination and memory kinetics after influenza vaccination. Violin plots with median and interquartile range (IQR). Statistics: AICOVI data: Mixed-effects ANOVA with Geisser-Greenhouse correction for 13 a *priori* selected comparisons (as described in the methods section), followed by Sidak's multiple group comparisons. Only significantly different groups are indicated. AIGI data: Friedman test with Dunn’s test for multiple comparisons. *p ≤ 0.05; **p ≤ 0.01; ***p ≤ 0.001; ****p ≤ 0.0001. Bmem, memory B cells; MENSA, medium enriched for newly synthesized antibodies. Panel **(A)** was created with BioRender.com.

To analyze the kinetics of the antibody and B cell response to vaccination, we compared two opposing immunological scenarios. COVID-19 vaccination provided us with the unique possibility to study the immune response to prime-boost-(boost) vaccination in a naïve population (AICOVI study, **Figures 1**, **2A**). The influenza vaccination is a perfect example of a boost immunization in a population that is largely primed due to prior vaccination and/or influenza infection (AIGI study, **Figure 2A**).

To monitor the plasma IgG-, plasmablast- and Bmem responses to COVID-19 vaccination, we complemented a serological assay with an *ex vivo* B cell cultivation approach. A total of 46 healthy hospital employees without prior SARS-CoV-2 infection who received either homologous or heterologous regimens of COVID-19 vaccination (AICOVI study) were analyzed: Fourty-three subjects received two vaccinations; 29 also received a 3^rd^ vaccination. 32 subjects received two doses of BNT, of which 19 subjects also received a third dose of BNT. The remaining subjects (n = 11) received heterologous vaccination schemes with AZD, BNT or MOD (**Figure 1**; **Supplementary Table 1**; **Supplementary Figure 1**). Time intervals between the 1^st^ and 2^nd^ vaccination were 26–29 days and 84–85 days for the homologous BNT and heterologous vaccination schemes, respectively (**Figure 1**; **Supplementary Figure 1**). Plasma and PBMCs were obtained from whole blood samples on the days of the 1^st^, 2^nd^ and 3^rd^ vaccinations, as well as 7 and 14 days later.

Coronavirus-specific IgG antibodies were quantified using an in-house bead-based multiplexed immunoassay (Corona Array). It contained six SARS-CoV-2 antigens: the S1 subunit, the RBD domain, the S2 subunit, and S prefusion as well as NP of the original SARS-CoV-2 strain Wu01; the S1 subunit of the Alpha variant (B.1.1.7); and the S1 subunits of the four endemic HCoVs (HCoV-229E, -HKU1, -NL63 and -OC43) (**Supplementary Table 2**). The Corona Array was validated using 10 sera from hospitalized convalescent COVID-19 patients and 20 sera from healthy controls obtained in the pre-COVID-19 era (**Supplementary Figure 2**). It was robust and reliable, with the coefficients of variation for the individual antigens ranging between 10.4% and 20.2% (based on 73 technical replicates of a plasma pool).

### 3.1 Prime-boost kinetics of specific plasma IgG, plasmablasts and Bmems after COVID-19 vaccination

Before vaccination, the AICOVI subjects had no plasma IgG antibodies against the SARS-CoV-2 S1, confirming that they were immunologically naïve (**Figure 2B**; for individual time courses see **Supplementary Figure 3**). The 1^st^ vaccination (v1) induced a strong IgG response in 43/44 subjects at day 14 (d14) post-immunization (median v1d14/v1d0 ratio: 595.5; **Supplementary Table 3**). Antibody levels were further enhanced in all subjects upon the 2^nd^ vaccination (median v2d14/v1d14 ratio: 32.4). Notably, the antibody response was much faster after the 2^nd^ vaccination (peak at d7) than after the 1^st^ vaccination (peak at d14). Six to ten months after the 2^nd^ vaccination, plasma IgG levels against S1 had decreased by an average factor of 24.1 (median v3d0/v2d14 ratio). However, the 3^rd^ vaccination restored the antibody levels to peak levels comparable to those observed shortly after the 2^nd^ vaccination.

To monitor the plasmablast surge following vaccination and to assess their specificity and functionality, we cultured PBMCs and analyzed the culture supernatants for spontaneously secreted antibodies (medium enriched for newly synthesized antibodies, MENSA). Fourteen days after the 1^st^ vaccination, anti-SARS-CoV-2-S1 IgG antibodies became measurable in MENSA samples from almost all probands (42/44) (**Figure 2C**). After the 2^nd^ and 3^rd^ vaccinations, the plasmablast response became faster and stronger, and the spontaneous IgG secretion peaked already at d7. This can be explained by Bmems, which had developed in the meantime (see below) and can differentiate into plasmablasts more quickly than naïve B cells. The peak levels of specific antibodies in MENSA increased by a factor of 32.4 after the 2^nd^ vaccination (median v2d14/v1d14 ratio) but did not increase further after the 3^rd^ vaccination, indicating similar numbers of circulating plasmablasts after the 2^nd^ and 3^rd^ vaccinations. Notably, the plasmablast activity in the blood did not return to baseline levels during the observation periods after the 1^st^ and 2^nd^ vaccinations (median IgG binding: 0.04 AU (v1d0), 0.25 AU (v2d0), and 1.61 AU (v3d0); **Supplementary Table 4**), demonstrating an ongoing immune response.

To assess Bmems, we stimulated PBMCs with interleukin 2 and the Toll-like receptor 7/8 ligand R848, which induces their differentiation into antibody-secreting plasmablasts. MENSA+ supernatants therefore contain antibodies produced by Bmem progeny in addition to those derived from circulating plasmablasts (MENSA). No S1-specific Bmem activity was observed before and on d7 after vaccination, emphasizing that all subjects were immunologically naïve to SARS-CoV-2. 14 days after the 1^st^ vaccination, MENSA+ samples contained almost 10 times higher concentrations of S1-specific IgG than the matched MENSA samples (median MENSA+/MENSA ratio: 8.6) (**Figure 2D**), implying that activated Bmems dominated the antibody response in MENSA+ already at this early timepoint. The difference between MENSA+ and MENSA was most striking on the day of the 2^nd^ vaccination (v2d0), when spontaneous antibody secretion had almost ceased, whereas the antibody concentration in MENSA+ had increased (median MENSA+/MENSA ratio: 511.6). This shows that many Bmems had developed in the period between d14 and d24–85 after the 1^st^ vaccination. After the 2^nd^ and 3^rd^ vaccinations, S1-specific antibody levels peaked at d7, showing that Bmems expanded in response to restimulation. This was much faster than Bmem generation from naïve B cells. Six to ten months after the 2^nd^ vaccination, Bmem-derived antibody levels against S1 had decreased by factor of 15.1 (median v3d0/v2d14 ratio). The 3^rd^ vaccination restored them to the level observed 7 days after the 2^nd^ vaccination. To conclude, the kinetics of the plasma IgG-, plasmablast- and Bmem-responses to COVID-19 vaccination reflected the kinetics of a prime-boost immunization of naïve individuals.

During the course of the AICOVI study the recommendations and availability of COVID-19 vaccines were changing which led to different vaccination schemes within the study (see also **Figure 1**) (3). A comparison of the two largest subgroups (BNT/BNT/BNT, AZD/BNT/BNT) showed that the kinetics of the anti-S1 antibody and B cell response were similar in both groups (**Supplementary Figure 4**). The magnitude of plasma antibody and B cell response, however, was slightly enhanced in the heterologous vaccination group after the 2^nd^ vaccination.

### 3.2 Memory kinetics of specific plasma IgG, plasmablasts and Bmems after influenza vaccination

We then contrasted our results on COVID-19 vaccination in naïve subjects with the effect of a single influenza vaccination in a primed cohort (AIGI study) (**Figure 2A**). Subjects received Vaxigrip Tetra^®^ 2020/2021, a quadrivalent split-virion, inactivated vaccine. The B cell responses were analyzed as in the AICOVI study. Already before vaccination, all subjects had high serum IgG antibodies to the influenza vaccine (median: 11,018 AU), which increased only moderately after vaccination, 1.3-fold on d7 and 1.7-fold on d14 (median ratio, **Figure 2E**). Influenza-specific plasmablasts were absent before vaccination, emerged on d7 on their transit to the bone marrow and disappeared again on d14 after vaccination (**Figure 2F**). In contrast, most subjects harbored Bmems to the influenza vaccine prior to vaccination. Vaccination boosted this arm of the vaccine-specific memory by a factor of 8.3 (median d14/d0 ratios) (**Figure 2G**). Notably, the serum IgG- and Bmem-responses to the influenza vaccine peaked already at d7, in agreement with the notion that secondary immune responses are faster than the primary response.

Overall, these data clearly illustrate two poles of vaccine-induced immune responses. At one end of the spectrum, the COVID-19 vaccinees were immunologically naïve and responded with a slow and moderate primary response, followed by a faster and stronger secondary response. Waning antibody levels and Bmem responses several months after the 2^nd^ vaccination were restored by the 3^rd^, i.e., booster vaccination. At the other end of the spectrum, the influenza vaccinees possessed robust immune memory for the vaccine antigens already before vaccination. Vaccination boosted antibody production and Bmem numbers.

### 3.3 The 3^rd^ vaccination dose elicits neutralizing plasma antibodies against the SARS-CoV-2 Omicron variant

**Figure 3:**
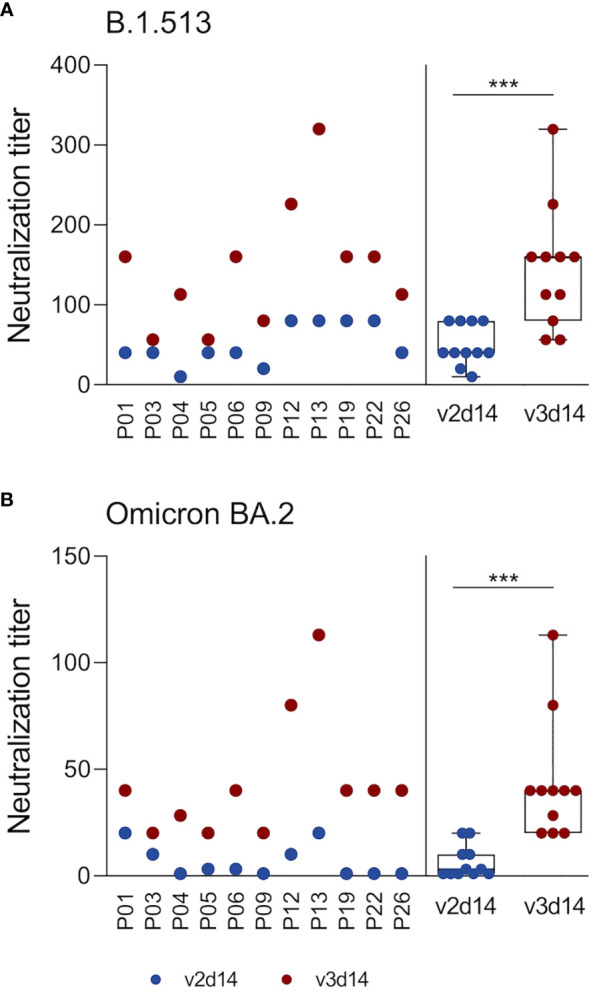
3^rd^ SARS-CoV-2 vaccination elicits neutralizing capacity against the SARS-CoV-2 Omicron BA.2 variant. Neutralizing plasma antibodies against SARS-CoV-2 were determined in an in-house Vero cell-based virus neutralization test. Analyses were restricted to plasma samples retrieved 14 days after 2^nd^ and 3^rd^ vaccinations from 11 subjects who received 3 doses of the BNT vaccine. Serially diluted plasma samples were co-incubated with an original B.1.513 strain (isolated in spring 2020 in Germany, pre-VOC) **(A)** or an Omicron BA.2 strain **(B)** on Vero cells for 4-6 days. Virus-induced cytopathic effects were determined by crystal violet staining. The neutralization titer denotes the highest plasma dilution that prevented the formation of cytopathic effects. Statistics: Wilcoxon matched pairs signed rank test. ***p ≤ 0.001. Abbreviations: v2d14, 14 days after the 2^nd^ vaccination; v3d14, 14 days after the 3^rd^ vaccination.

Next, we examined whether vaccine-induced antibodies can neutralize a type B.1.513 strain (2020, pre-VOC) (38), as well as an Omicron BA.2 variant in a Vero cell-based virus neutralization assay. We focused our analyses on plasma samples obtained 14 days after the 2^nd^ and 3^rd^ vaccination and on a representative AICOVI subset of 11 vaccinees, who received homologous BNT/BNT/BNT vaccinations with complete sample sets. This BNT subgroup was not statistically different from the whole AICOVI cohort in terms of S1 antibody binding (**Supplementary Figure 5**), as well as sex, age, and BMI. Neutralizing capacity against the B.1.513 strain was detected in all subjects after the 2^nd^ dose (median neutralizing antibody titer: 40) and was strongly enhanced after the 3^rd^ dose (median neutralizing antibody titer: 160) (**Figure 3**). This is in contrast to the results of our binding assay, which showed that the S1-binding antibody levels were not higher after the 3^rd^ dose than after the 2^nd^ dose. Neutralizing antibodies against BA.2 were only sporadically detected after the 2^nd^ vaccination, but the 3^rd^ vaccination elicited neutralizing plasma antibodies against BA.2 in all individuals (median neutralizing antibody titer: 40).

### 3.4 COVID-19 vaccination-induced antibody and B cell responses do not cross-react with S1 from endemic HCoVs

**Figure 4:**
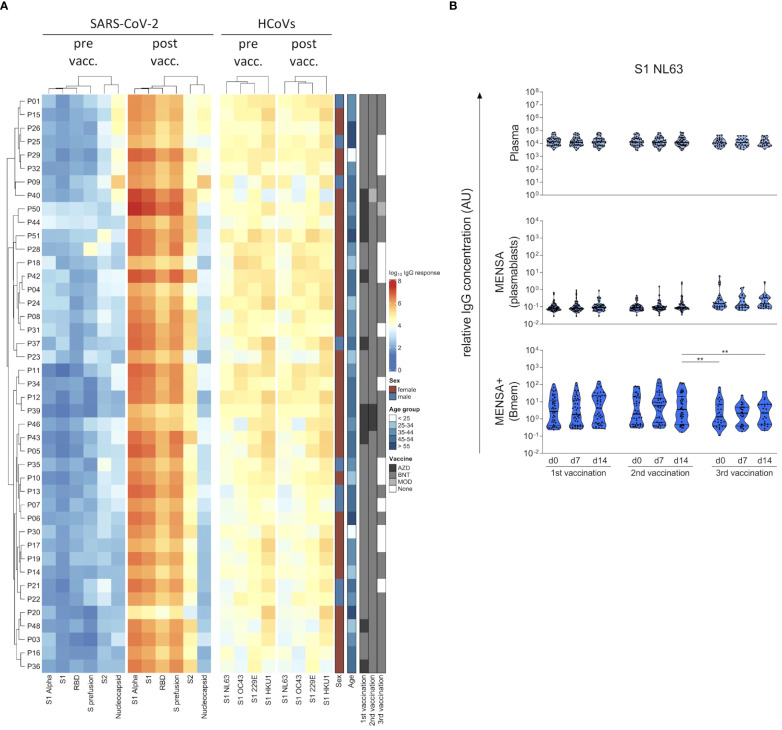
COVID-19 vaccination-induced B cell response discriminates between the Spike S1 subunit from SARS-CoV-2 and endemic human coronaviruses (HCoVs). Plasma IgG binding to six SARS-CoV-2 antigens (Spike S1 subunit, receptor binding domain (RBD), S2 subunit, S prefusion and NP from the original strain Wu01, and S1 from the Alpha variant (B.1.1.7)) as well as the spike S1 subunit from the four endemic HCoVs (HCoV-229E, -HKU1, -NL63 and -OC43) were determined at the day of 1^st^ COVID-19 vaccination (pre vaccination, pre vacc.) as well as 14 days after the 2^nd^ vaccination (post vaccination, post vacc.) using a bead-based Corona Array and visualized in a heat map **(A)**. Details on the sex, age and vaccination schemes of the subjects are provided. IgG antibodies against HCoV-NL63 S1 were quantified in plasma, MENSA, and MENSA+ samples obtained over the course of SARS-CoV-2 vaccination **(B)**. Violin plots depict the median and IQR. The COVID-19 vaccination-induced B cell response is highly specific for S1 from SARS-CoV-2, showing no cross-reactivity with S1 from endemic HCoVs. Statistics: Mixed-effects ANOVA with Geisser-Greenhouse correction for 13 *a priori* selected comparisons (as described in the methods section), followed by Sidak's multiple group comparisons. **p ≤ 0.01. AZD, ChAdOx1-S (Vaxzevria^®^; AstraZeneca); BNT, BNT162b2 (Comirnaty^®^; BioNTech/Pfizer); MOD, Spikevax^®^ (Moderna); vacc, vaccination.

The SARS-CoV-2 S protein shares around 30% sequence identity with the S proteins of the endemic HCoVs, with the S2 subunit being more conserved than S1 (30). To assess whether B cells can discriminate between SARS-CoV-2 and HCoVs, we compared antibody levels in plasma and MENSA samples against S1 from the original SARS-CoV-2 strain Wu01 versus the common cold HCoVs 229E, HKU1, NL63 and OC43 (**Figure 4**). Prior to COVID-19 vaccination, all subjects harbored moderate plasma IgG antibody levels against the S1 subunit of all four endemic HCoVs, which did not change upon vaccination (**Figure 4A**). In contrast, plasma antibodies against SARS-CoV-2 S1 antigen were at background level before the first vaccination (v1d0) and increased by more than four log units by v2d14 (median v2d14/v1d0 ratio: 22,520). Antibody levels against the S1 subunit from SARS-CoV-2 versus HCoVs were not correlated with each other (**Supplementary Figure 6**).

Next, we determined antibody binding to S1 from all four HCoVs in MENSA and MENSA+ samples to estimate cross-reactivity with SARS-CoV-2 at the B cell level. **Figure 4B** exemplifies the results for HCoV-NL63, data on the other three endemic viruses are compiled in the **Supplementary Figure 7**. Already at baseline we observed a strong pre-existing Bmem response. While plasma antibody levels against NL63 were quite uniform, ranging from 3168 to 74,012 AU (v1d0, factor: 22), IgG levels in MENSA+ samples suggested that Bmem frequencies in peripheral blood were much more variable (v1d0: 0.2–110 AU; factor: 474). Circulating HCoV-specific plasmablasts were only rarely detected. In summary, the vaccination-induced antibody and B cell responses to the diverse S1 subunit were specific for SARS-CoV-2.

### 3.5 Antibodies from endemic HCoVs cross-react with S2 and NP, but not with S1

**Figure 5:**
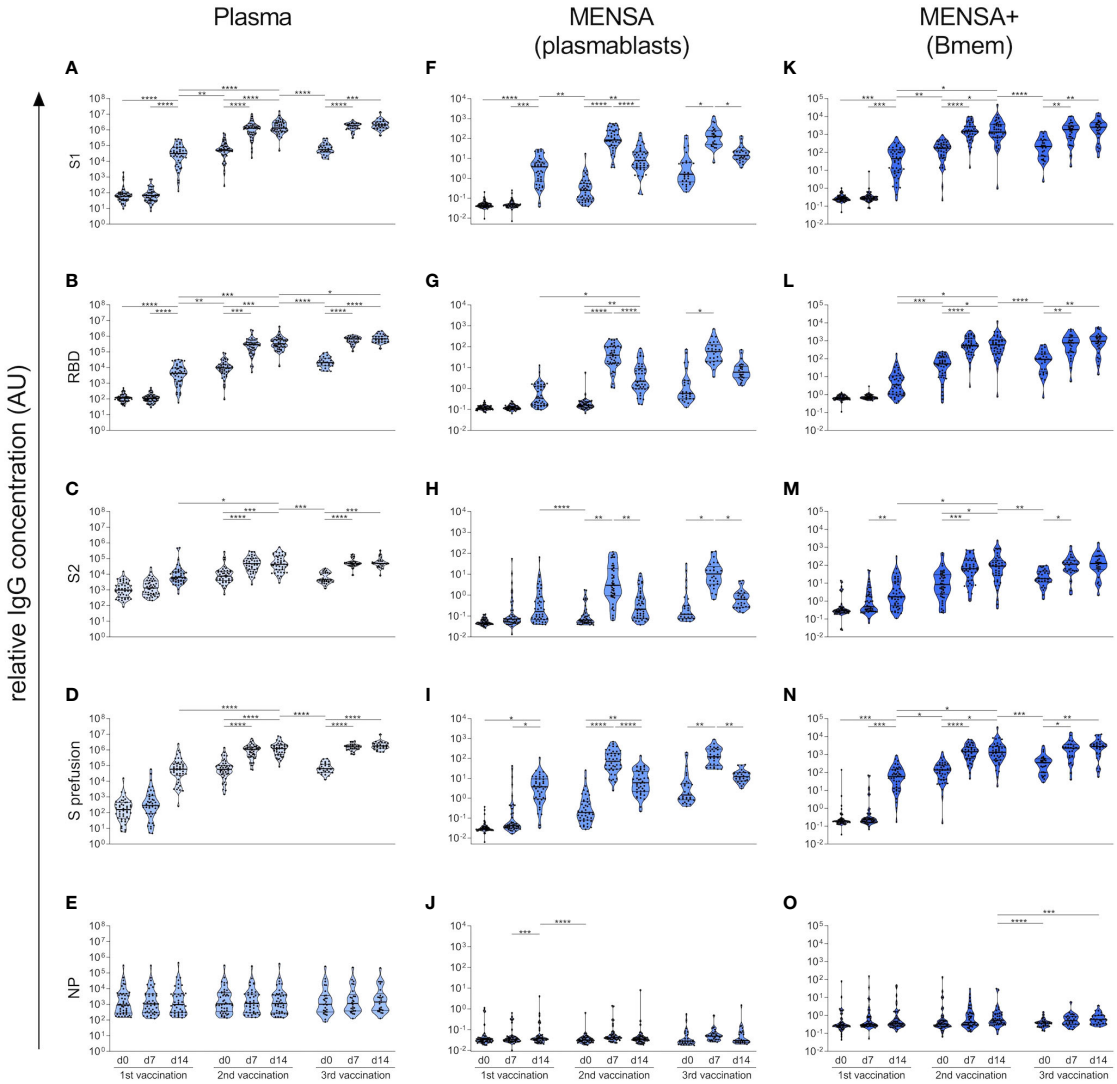
Vaccination-induced B cell response predominantly targets SARS-CoV-2 S1. Using a bead-based Corona Array, IgG antibodies against S1 subunit, RBD domain, S2 subunit, spike prefusion as well as NP from SARS-CoV-2 (original strain Wu01) were quantified in plasma **(A–E)**, MENSA **(F–J)**, and MENSA+ **(K–O)** samples obtained over the course of COVID-19 vaccination. Vaccination-induced antibody and B cell responses predominantly target SARS-CoV-2 S1, while the cross-reactive epitopes on S2 were less immunogenic. Violin plots depict the median and IQR. Statistics: Mixed-effects ANOVA with Geisser-Greenhouse correction for 13 a priori selected comparisons (as described in the methods section), followed by Sidak's multiple group comparisons. *p ≤ 0.05; **p ≤ 0.01; ***p ≤ 0.001; ****p ≤ 0.0001.

The SARS-CoV-2 S protein is a homotrimer, with each monomer consisting of two subunits, S1 and S2 (44). The S1 subunit contains the RBD responsible for binding to the host cell receptor angiotensin converting enzyme 2, while the S2 subunit mediates fusion between the viral and host cell membranes (44). To investigate which region of the S protein is predominantly targeted by the antibody response, we compared antibody binding to S1, RBD, S2, and S prefusion (spike ectodomain, stabilized prefusion conformation) as well as NP in plasma (**Figures 5A–E**), MENSA (**Figures 5F–J**), and MENSA+ (**Figures 5K–O**). Before vaccination, subjects lacked plasma antibodies against S1 and RBD (median IgG binding of 59.6 AU and 114.9 AU, respectively), but showed low levels of plasma antibodies against S2 (median: 976.4 AU) (**Figures 5A–C**), suggesting some cross-reactivity of this subunit with the endemic HCoVs. Similarly, anti-S2 IgG was detected in a few MENSA+ samples before vaccination, pointing toward cross-reactive Bmems (**Figure 5M**).

As described above, vaccination with the S protein of SARS-CoV-2 induced a very strong immune response against the S1 subunit of this virus but did not influence the humoral response to S1 of the endemic HCoVs. The magnitude and kinetics of the antibody, plasmablast and Bmem responses to SARS-CoV-2 S1, RBD, and S prefusion were highly concordant due to shared epitopes (**Figures 5A, B, D, F, G, I, K, L, N**). Accordingly, we observed a very strong correlation between IgG binding to S1, RBD and S prefusion (Spearman correlation coefficients > 0.96) (**Supplementary Figure 6**). In contrast, the S2 subunit appeared to be less immunogenic, inducing only a moderate increase in plasma IgG binding with each vaccination (6.3-fold (v1d14/v1d0), 4.8-fold (v2d14/v2d0), and 10.1-fold (v3d14/v3d0) increase) (**Figure 5C**). Similarly, anti-S2 antibody levels were lower in the MENSA and MENSA+ samples throughout the observation period, implying a lower frequency of S2-specific plasmablasts and Bmems (**Figures 5H, M**). In line with the pre-existing, cross-reactive plasma antibodies against S2, our MENSA data indicate a rapid re-activation of S2-specific Bmems and differentiation into plasmablasts in some individuals after the 1^st^ vaccine dose, reflecting a secondary immune response (**Figure 5H**).

The antibody and B cell response to NP also demonstrated cross-reactivity. Already before vaccination, low-level antibodies against NP were detected in plasma samples from all subjects (median: 905.3 AU) (**Figure 5E**), as well as in some MENSA+ samples (**Figure 5O**). As anticipated, the plasma IgG levels against NP were not affected by vaccination.

Previous studies have reported, that HCoV-induced memory B cell responses might inhibit the response to novel SARS-CoV-2 antigens or epitopes after vaccination or infection (45, 46). To test for this so-called imprinting or original antigenic sin effect, we correlated the pre-existing antibody- and Bmem levels against HCoV S1 with the magnitude of the vaccination-induced antibody response to SARS-CoV-2 antigens. High antibody titers and Bmem levels against HCoV S1 did not affect the *de novo* generation of SARS-CoV-2-specific antibodies at all (i.e., against S1, RBD, S2 and NP) (**Supplementary Figure 8**).

Taken together, our results clearly show that the vaccination-induced humoral immune response predominantly targets the S1 subunit of the SARS-CoV-2 spike protein, whose epitopes are novel to the immune system, while the cross-reactive epitopes on the S2 subunit are less immunogenic.

## 4 Discussion

COVID-19 vaccination generates protective immunity by inducing potent antibody responses as well as Bmems, which are rapidly activated and produce new antibodies upon antigen re-exposure. While the frequency of S-specific plasmablasts and Bmems after prime and boost vaccinations against COVID-19 has been well documented (7–9, 13, 26, 47), our knowledge of the functional capacity of plasmablasts and Bmems has been limited. We have profiled the generation of S-specific antibodies and assessed the functional capacity of vaccine-induced plasmablasts and Bmems in naïve adults who were immunized with three doses of a COVID-19 vaccine. This longitudinal study enabled a detailed analysis of the magnitude, durability and quality of SARS-CoV-2 vaccine-induced humoral immunity over almost a year and multiple vaccine doses. A comparison with influenza vaccination, a prototypical booster vaccination, illustrated the differences in the vaccine responses between naïve and primed individuals. Moreover, our multiplex-approach provided insights into the specificity of the B cell response and its cross-reactivity with seasonal HCoVs.

Our approach of combining classical serology with MENSA illustrates the kinetics of vaccine-induced antibody and B cell responses particularly well, and highlights the marked difference in the kinetics between COVID-19 and influenza vaccination. The influenza vaccination elicited a typical secondary immune response, with a moderate boost of vaccine-specific serum IgG levels and Bmem responses 7 days post vaccination that coincided with a transient plasmablast peak, as previously reported (40, 48, 49). On the other hand, after COVID-19 vaccination, the response of plasma antibodies, circulating plasmablasts and Bmems against the SARS-CoV-2 S1 reflected the typical kinetics of a prime-boost immunization in a naïve cohort. Besides the immunization status (naïve versus primed), both cohorts also differed in the employed vaccine type (mRNA vaccine versus inactivated vaccine), which likely also had a moderate effect on the strength of the induced humoral immune response (50, 51).

The 1^st^ COVID-19 vaccine dose induced a primary immune response. In detail, vaccination induced a robust S1-specific IgG response in all subjects at 14 days, coinciding with a short-termed appearance of S1-specific plasmablasts in the peripheral blood (MENSA) and the induction of specific Bmems (MENSA+). While the short-lived plasmablasts disappeared rapidly in the periphery, antibody levels after the 1^st^ vaccination remained stable for several weeks, as they get replenished by long-lived plasma cells (12, 52). These kinetics are supported by other vaccination studies using ELISpot or flow cytometry-based approaches (8, 9, 15, 16, 40, 41).

The 2^nd^ and 3^rd^ COVID-19 vaccinations triggered a secondary immune response. IgG, plasmablast and Bmem responses were much faster (7 versus 14 days) and stronger than after the priming dose. Similar antibody and Bmem kinetics were observed by other research groups (7–9, 13, 53). This effect is attributed to the reactivation of vaccine-specific Bmems, which are present in much higher numbers and also get more easily activated than naïve B cells. Vaccine-specific Bmems undergo further affinity maturation and differentiate into antibody-secreting plasmablasts. Therefore, secondary immune responses are generally characterized by a faster and stronger antibody response and the generation of high affinity antibodies.

Six to ten months after the 2^nd^ vaccination, S1-specific plasma antibody levels as well as Bmems (MENSA+) had decreased by a factor of 24.1 and 15.1, respectively, which is in line with other reports (16, 17, 53). Long-term studies revealed that vaccine-induced spike-specific antibody kinetics follow a bi-phasic decline, with an initial rapid decay (half-life: 28 days) followed by a stabilization of antibody levels accompanied by a continuous improvement of the virus-neutralizing potency (15–17, 54). In line with this, a multiparametric flow cytometry-based study by Goel et al. revealed an initial decline in S1-specific class-switched Bmem frequencies after a 2^nd^ COVID-19 vaccination, followed by a stabilization for the next 8 months (16). A similar antibody waning was also observed after the 3^rd^ dose (16, 17), emphasizing that these kinetics mirror the dynamics of a typical immune response following vaccination.

Apart from replenishing anti-S1 antibody levels, the 3^rd^ vaccination has two decisive advantages: (1) enhanced neutralizing potency against the vaccine antigen and (2) the generation of broadly neutralizing antibody responses that are also effective against more recent SARS-CoV-2 variants. We detected neutralizing plasma antibodies against the original (pre-VOC) B.1.513 strain in all tested subjects after the 2^nd^ vaccination, which were strongly enhanced upon the 3^rd^ vaccination as previously reported (16, 53). Thus, while total anti-S1 IgG levels appeared to plateau, their neutralizing potency strongly improved. Similarly, others reported that total SARS-CoV-2-specific antibody levels waned over time after the 2^nd^ dose, while neutralizing antibody levels and protection against hospitalization and death persisted at high levels for at least six months (8, 12, 16).

The second benefit of booster vaccinations is the expansion of neutralizing capacity toward newer variants. Here, we focused on the Omicron BA.2 variant as previous studies already demonstrated that immune escape is much more pronounced with Omicron than with the Beta or Delta variants (53, 55). While all individuals harbored neutralizing antibodies against the original B.1.513 strain after the 2^nd^ vaccination, only few subjects had neutralizing antibodies against Omicron BA.2. After the 3^rd^ vaccination, however, all individuals developed BA.2-neutralizing antibodies, but antibody levels were on average 3-fold lower than those against the B.1.513 strain. These results reflect an expansion of the antibody repertoire after the 3^rd^ vaccination, despite being vaccinated with the original mRNA vaccine, and are consistent with other studies (16, 24, 33, 35, 53, 55, 56). These broadly neutralizing antibody responses are likely mediated by the generation and expansion of B cell clones expressing broadly reactive and potent antibodies (13, 21, 53, 57–62). Even though the generation of such broadly neutralizing antibodies is advantageous, their levels are often insufficient to prevent breakthrough infections (63). Indeed, individuals with hybrid immunity (i.e. immunity developed by a combination of SARS-CoV-2 infection and vaccination) seem to be better protected against COVID-19-related hospitalizations or severe disease than individuals who were infected or vaccinated alone (64). Our study therefore supports the utility of a 3^rd^ vaccine dose to recall immunological memory and replenish anti-S antibody levels to provide immediate protection upon SARS-CoV-2 exposure. Moreover, booster vaccinations promote continuous antibody maturation in germinal centers resulting in an enhanced neutralizing potency against the vaccine antigen and, more importantly, the generation of broadly neutralizing antibodies that are effective against newer variants. More research is needed to study the effects of variant-adapted vaccines and elucidate how boosting with a modified antigen enhances recall responses in comparison to boosting with the original antigen.

Subjects receiving a heterologous (AZD/BNT/BNT) vaccination scheme tended to have a stronger antibody and B cell response than those receiving a homologous (BNT/BNT/BNT) vaccination scheme. Several other studies compared the plasma IgG binding following BNT/BNT and AZD/BNT vaccination regiments, with partially contradicting findings (65–67). Overall, both BNT/BNT and AZD/BNT induce strong IgG binding and neutralizing responses and are clearly superior to the AZD/AZD vaccination scheme. A systematic review and a metaanalysis suggest that heterologous AZD/BNT vaccination induces a comparable or slightly higher antibody response than the homologous BNT/BNT immunization (68, 69). While most other studies have obtained samples one or several months after vaccination (65–69), our study provides information on the early antibody response to homologous versus heterologous vaccination, i.e. 7 and 14 days post vaccination. Moreover, our data suggest that not only the antibody response but also the underlying functional plasmablast and Bmem responses tend to be stronger after heterologous as compared to homologous vaccination.

In addition to studying SARS-CoV-2-specific antibody and B cell responses, we also analyzed the pre-existing humoral immunity against HCoVs and its effects on the vaccine response. In line with other studies (70, 71), we observed 100% seroprevalence against all four seasonal HCoVs prior to COVID-19 vaccination. This pre-existing immunity to HCoVs showed some cross-reactivity with the SARS-CoV-2 spike S2 subunit, but not the S1 subunit, which can be explained by a higher conservation of B cell epitopes in S2 as compared to S1 across coronavirus species (21). In our study, the baseline IgG levels against SARS-CoV-2 S2 were around 10-fold higher than those against S1 and RBD, which is in accordance with previous studies (15, 71–73). Moreover, our MENSA data indicate that in some individuals the S2-specific Bmems are rapidly activated to differentiate into plasmablasts after the 1^st^ vaccine dose, indicating a secondary immune response. In line with our data, single-cell transcriptomics of pre- and post-vaccination samples revealed a rapid onset of the S2-specific IgG and plasmablast response, whereas the S1/RBD-specific B cell response showed the kinetics of a primary response (21).

Previous studies have demonstrated that both vaccination and COVID-19 infection can induce antibodies and plasmablasts that cross-react with the S2 subunit of HCoVs, most commonly OC43 and HKU1 (21, 25–27, 45, 46). Probably, cross-reactive Bmems had been generated during respiratory tract infections with HCoVs and were re-activated upon challenge with the SARS-CoV-2 S protein. However, the clinical relevance of these cross-reactive antibodies is likely to be limited, as pre-pandemic SARS-CoV-2 reactive antibodies were not associated with protection against SARS-CoV-2 infection or hospitalization due to COVID-19 (71).

Conversely, we observed no cross-reactivity between the highly variable S1 subunits of SARS-CoV-2 and HCoVs. COVID-19 vaccination did neither affect the antibody levels against HCoV S1 subunits, nor did it induce HCoV-directed plasmablast and Bmem responses in any of the tested individuals. Consistent with our findings, Anderson et al. observed that the antibody levels against HKU1 and OC43 S1 were not affected by vaccination or infection (46). Similarly, vaccine-induced antibodies cross-reacting with the HCoV RBD/S1 have only rarely been reported (21).

Our findings also provide a new perspective on the “original antigenic sin” (OAS) hypothesis that is often discussed in the context of COVID-19 vaccination and infection. OAS refers to a hypothetical preference of the immune system to recall, or ‘back-boost’, existing memory B cells specific for epitopes shared by several antigens, rather than priming naїve B cells recognizing new epitopes when encountering a novel but closely-related antigen (74). In the context of COVID-19, it was postulated that pre-existing HCoV-specific B cell responses could inhibit the response to the new SARS-CoV-2 antigens or epitopes after vaccination or infection (73, 75). In the AICOVI study, we detected baseline antibody binding to the SARS-CoV-2 S2 subunit, which was indeed likely due to cross-reactivity with HCoV S2 subunits. COVID-19 vaccination, however, only moderately boosted antibodies against these conserved S2 epitopes (median ratio (v2d14/v1d0): 42.6), while inducing a very strong primary immune response against novel, non-cross-reactive S1 epitopes (median ratio (v2d14/v1d0): 22,520). This dichotomy, i.e. a moderate back-boost to S2 epitopes versus a pronounced primary response to S1 epitopes, was also observed in previous studies (26, 46). In addition, we showed that pre-existing high antibody titers and Bmem levels against HCoV S1 did not impact on the *de novo* generation of SARS-CoV-2-specific antibodies against S1, RBD, S2 and NP at all. Similarly, other studies observed no OAS effect for HCoV S1, and only a moderate OAS effect for HCoV S2 immune memory (26, 45, 46). To conclude, antibodies that bind to novel, non-cross-reactive epitopes dominate the humoral immune response to COVID-19 vaccination, while back-boosted S2-specific antibodies seem to play only a marginal role.

Our MENSA approach has some advantages over other commonly used methods for studying antibody and B cell responses. This *ex vivo* culture-based approach measures the ability of plasmablasts and Bmems to secrete S-specific antibodies, which are subsequently quantified in the culture supernatant. Antibodies in MENSA reflect antibody secretion by recently activated plasmablasts, while MENSA+ antibodies are predominantly released from re-activated Bmems (48, 76, 77). As antibody levels in MENSA correlate well with the total number of antibody secreting cells (as determined by ELISpot) (78), we employed MENSA as a surrogate marker for the number of vaccine-specific plasmablasts and Bmems. In contrast to classical serology, MENSA enables the evaluation of vaccine- or infection-induced antibody responses without the interference of pre-existing serum antibodies, and thus has a high diagnostic potential (78–80). Moreover, the MENSA approach assesses the functional capacities (i.e. antibody secretion) of plasmablasts and Bmems, while flow cytometry-based analyses and single cell sequencing only provide insights into the frequency of plasmablasts and Bmems (7–9, 21). Vice versa, our approach does not allow to quantify the antigen-specific plasmablasts or Bmems, and antibody levels in MENSA+ might be skewed by B cell proliferation. Another highly informative method is the enumeration of antibody secreting cells by ELISpot or FluoroSpot (41). The advantage of our MENSA approach, however, is that antibodies in MENSA remain available for subsequent analyses. Thus, the analysis of MENSA can provide unique insights into the functional capacity of plasmablasts and Bmems and complements established methods.

Our AICOVI and AIGI studies have some limitations. Our study solely focused on antibody, plasmablast and Bmem responses, while neglecting other immune cell types and soluble factors that modulate B cell differentiation and antibody secretion, such as follicular T helper cells, mesenchymal stem cells, cytokines, and chemokines (81–83). Moreover, immune memory is also mediated by vaccine-specific T cells, whose response has been deciphered in depth in other studies (35, 83). Another caveat is the variable sex composition of AICOVI and AIGI cohorts (78.3% vs. 52.9%). AICOVI probands were recruited without restrictions with regard to sex right at the start of the SARS-CoV-2 vaccination campaign which was initially prioritized for hospital employees with patient contact. The larger proportion of women among healthcare workers resulted in a surplus of female participants in the AICOVI study. While the majority of studies suggests that females have slightly (ca. 1.4-fold) higher antibody levels than males (84–86), others observed no difference or partially even higher responses in males (87, 88). Nonetheless, the reported small effect sizes are neglectable considering the strikingly different antibody responses induced by a single COVID-19 versus influenza vaccination (v1d14/v1d0 ratio: 595.5-fold and 1.7-fold). Moreover, the AICOVI subjects received different vaccination schemes as recommendations and availability of COVID-19 vaccines were changing in early 2021 (3). We analyzed the pooled data as ultimately both vector-based (AZD, MOD) and mRNA-based vaccines (BNT) rely on the S protein of the original SARS-CoV-2 virus. Another caveat concerns the biomaterial. While antibody levels in the AICOVI study were quantified in blood plasma, we used serum samples for the AIGI study. However, this most likely did not impact on our results as plasma and serum can be used interchangeably in antibody detection assays (89, 90). Finally, we performed our neutralization assays on a relatively small, but homogenous cohort: individuals with three BNT vaccinations with complete sample sets (n = 11). Since anti-S1 antibody binding, sex, age and BMI of these selected subjects were statistically representative for the entire cohort, we consider a selection bias very unlikely.

In summary, our findings highlight the importance of complementing classical serology with a functional evaluation of plasmablasts and Bmems, to gain a more detailed picture of the kinetics of COVID-19 vaccine-induced antibody and B cell responses. In contrast to previous studies that phenotyped B cell subtypes by flow cytometry or single cell sequencing, we used an *ex vivo* culture-based approach to gain insights into the capacity of plasmablasts and Bmems to secrete SARS-CoV-2-specific antibodies. Indeed, the immune response to priming and boosting differed strongly in their kinetics, strength and specificity. Our results encourage the application of booster vaccinations, since they not only replenish antibody levels, but also promote continuous antibody maturation resulting in an enhanced neutralizing potency against the vaccine antigen, and the generation of broadly neutralizing antibodies that are effective against newer variants. Finally, antibodies that bind to novel, non-cross-reactive S1 epitopes clearly dominate the humoral immune response to COVID-19 vaccination, while our data suggest that the often-discussed OAS plays only a minor role.

## Data availability statement

The raw data supporting the conclusions of this article will be made available upon request by the authors, without undue reservation.

## Ethics statement

The studies involving humans were approved by Ethics Committee of the University Medicine Greifswald. The studies were conducted in accordance with the local legislation and institutional requirements. The participants provided their written informed consent to participate in this study.

## Author contributions

KW: Conceptualization, Data curation, Formal analysis, Investigation, Methodology, Project administration, Supervision, Validation, Visualization, Writing – original draft, Writing – review & editing. KF: Conceptualization, Data curation, Formal analysis, Investigation, Methodology, Project administration, Supervision, Writing – review & editing. EA: Formal analysis, Investigation, Writing – original draft, Writing – review & editing. GA: Investigation, Writing – original draft, Writing – review & editing. CD: Data curation, Investigation, Methodology, Writing – review & editing. MM: Investigation, Writing – review & editing. RR: Investigation, Writing – review & editing. MV: Investigation, Writing – review & editing. AK: Investigation, Writing – review & editing. SM: Formal analysis, Software, Writing – review & editing. SW: Formal analysis, Visualization, Writing – review & editing. LU: Resources, Validation, Writing – review & editing. PF: Data curation, Formal analysis, Visualization, Writing – review & editing. HF: Funding acquisition, Supervision, Writing – review & editing. BB: Conceptualization, Funding acquisition, Project administration, Supervision, Writing – original draft, Writing – review & editing. DR: Conceptualization, Data curation, Investigation, Methodology, Project administration, Supervision, Validation, Writing – original draft, Writing – review & editing. SH: Conceptualization, Data curation, Formal analysis, Funding acquisition, Project administration, Supervision, Validation, Visualization, Writing – original draft, Writing – review & editing.
