# Supplementary material for: Non-cross-reactive epitopes dominate the humoral immune response to COVID-19 vaccination – kinetics of plasma antibodies, plasmablasts and memory B cells
Source: Front Immunol. 2024 May 14;15:1382911. doi: 10.3389/fimmu.2024.1382911 (PMC11130424; doi:10.3389/fimmu.2024.1382911)
Supplement: Supplementary file 1 [file DataSheet_1.pdf]

Supplementary Material

Non-cross-reactive epitopes dominate the humoral immune response to COVID-19 vaccination – kinetics of plasma antibodies, plasmablasts and memory B cells

Kilian A. Wietschel, Kevin Fechtner, Elmer Antileo, Goran Abdurrahman, Chiara A. Drechsler, Michelle Makuvise, Ruben Rose, Mathias Voß, Andi Krumbholz, Stephan Michalik, Stefan Weiss, Lena Ulm, Philipp Franikowski, Helmut Fickenscher, Barbara M. Bröker, Dina Raafat and Silva Holtfreter*

*** Correspondence:** silva.holtfreter@med.uni-greifswald.de

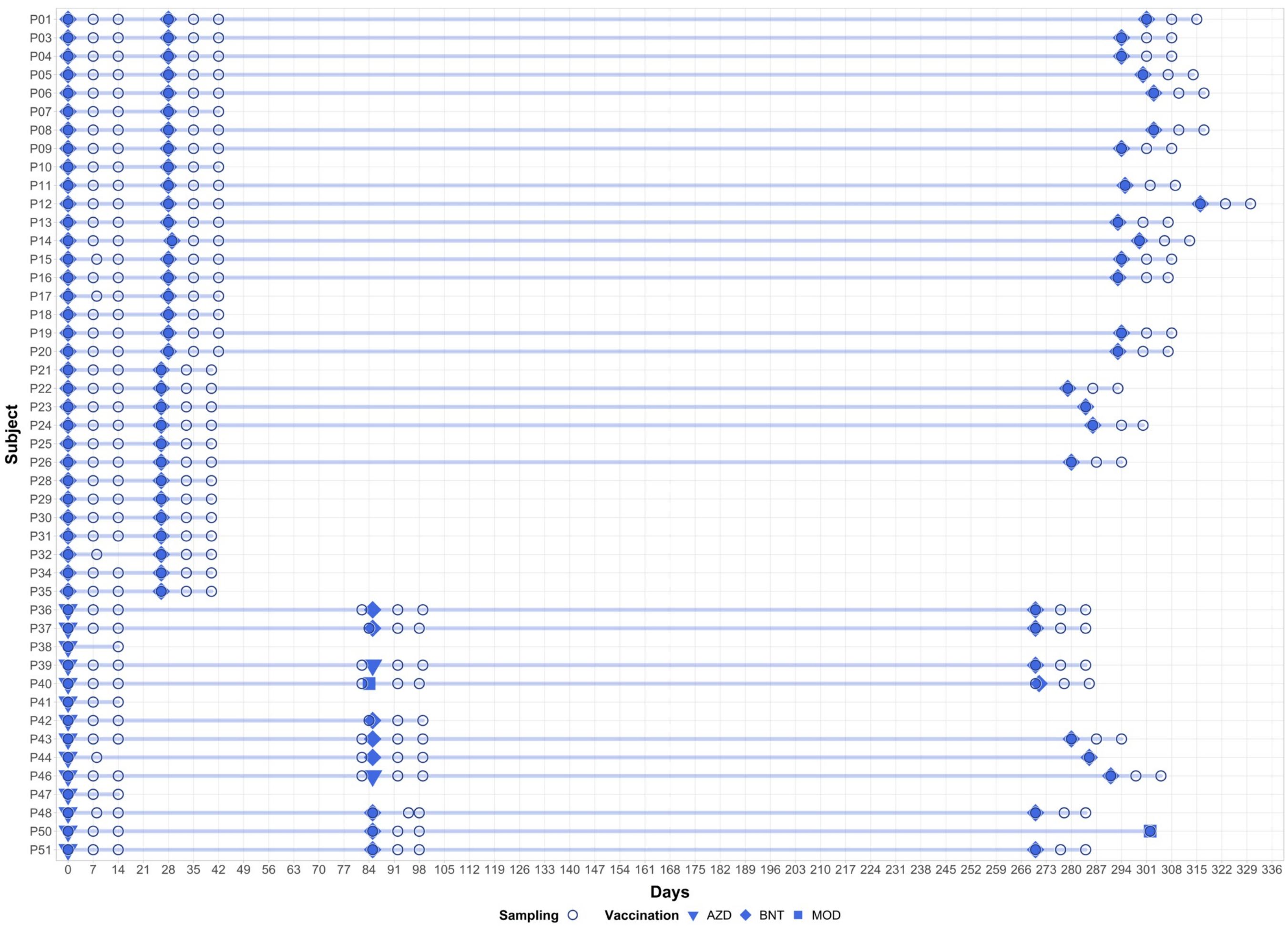

Supplementary Figure 1: Vaccination and sampling time points in the AICOVI study. AICOVI subjects (n = 46) received either a homologous or heterologous regimen of COVID-19 vaccines (see also Figure 1). Blood samples were obtained on the day of the 1st, 2nd and, for a subgroup of subjects, the 3rd COVID-19 vaccination, as well as 7 and 14 days later. *Abbreviations:* AZD, ChAdOx1-S (Vaxzevria®; AstraZeneca); BNT, BNT162b2 (Comirnaty®; BioNTec/Pfizer); MOD, Spikevax® (Moderna).

Supplementary Table 1: Cohort description of the AICOVI and AIGI studies

Study	Vaccination scheme	n		Age, mean ± SD [years][e]			Weight, mean ± SD [kg][e]			BMI, mean ± SD [kg/m²][e]		
		total	f (%)	total	f	m	total	f	m	total	f	m
AICOVI[a]	Homologous[c]	32	24 (75.0)	42.1 ± 11.1	40.5 ± 11.6	46.8 ± 8.6	76.8 ± 20.4	69.8 ± 14.7	97.8 ± 21.6	25,60 ± 5.4	24,6 ± 5.2	28,7 ± 5.0
	Heterologous[d]	14	12 (85.7)	43.2 ± 11.1	44.2 ± 9.6	37.0 ± 22.6	82.2 ± 19.7	81.6 ± 20.7	86.0 ± 18.4	28,10 ± 6.9	28,5 ± 7.3	25,0 ± 4.3
	total	46	36 (78.3)	42.4 ± 11.0	41.8 ± 11.0	44.8 ± 11.5	78.4 ± 20.2	73.7 ± 17.6	95.4 ± 20.7	26,40 ± 5.9	25,9 ± 6.2	28,1 ± 4.8
AIGI[b]	VaxigripTetra®	17	9 (52.9)	35.3 ± 11.8	39.3 ± 15.0	30,80 ± 3.8	68.6 ± 13.2	60.6 ± 10.9	79.0 ± 7.2	23,10 ± 3.3	21,9 ± 3.6	24,8 ± 2.0

[a]Subjects included in the AICOVI study (N = 46) received either homologous (n = 32) or heterologous (n = 11) regimens of COVID-19 vaccination.

[b]Subjects included in the AIGI study (N = 17) were vaccinated against seasonal influenza with VaxigripTetra® 2020/2021 (Sanofi Pasteur Europe).

[c] Subjects received 2 (n = 13) or 3 doses (n = 19) of BNT162b2 (BNT; Comirnaty®; BioNTec/Pfizer).

[d] 11 subjects received heterologous vaccination schemes with AZD1222 (AZD), BNT or Spikevac® (MOD). Another three subjects received only a single dose of AZD.

[e]Biometric data (sex, age, weight and BMI) were collected by standardized questionnaires on the day of the first vaccination and again on the day of the third vaccination with no relevant alterations (data not shown).

Abbreviations: AICOVI, Adaptive Immune Response after COVID-19 Vaccination; AIGI, Adaptive Immune Response after Seasonal Influenza Vaccination; BMI, body mass index

Supplementary Table 2: Recombinant proteins used in the bead-based Corona Array.

	Recombinant protein / protein domain (His-tagged)	Abbreviation	Company	Catalog Number
**SARS-CoV-2 Wuhan strain (Wu01, original strain)**				
1	Spike S1 subunit	S1	Sino Biological	40591-V08H
2	Spike RBD domain	RBD	Sino Biological	40592-V08H
3	Spike S2 subunit	S2	Sino Biological	40590-V08B
4	Spike prefusion conformation[a]	S prefusion	R&D Systems	10549-CV-100
5	Nucleocapsid	NP	Sino Biological	40588-V08B
**SARS-CoV-2 Alpha variant (B.1.1.7)**				
**6**	Spike S1 Alpha[b]	S1 Alpha	Sino Biological	40591-V08H12
**Endemic human coronaviruses (HCoVs)**				
**7**	HCoV-229E Spike S1 subunit	S1 229E	Sino Biological	40601-V08H
**8**	HCoV-HKU1 Spike S1 subunit	S1 HKU1	Sino Biological	40021-V08H
**9**	HCoV-NL63 Spike S1 subunit	S1 NL63	Sino Biological	40600-V08H
**10**	HCoV-OC43 Spike S1 subunit	S1 OC43	Sino Biological	40607-V08H1

[a]Ectodomain, stabilized prefusion conformation, resistant to furin cleavage; Val16-Lys1211 with a C-terminal His tag. Stabilizing mutations K986P and V987P promote the prefusion conformation. Two mutations, R682S and R685S, eliminate a furin protease cleavage site.

[b]Alpha variant, B.1.1.7: HV69-70 deletion, Y144 deletion, N501Y, A570D, D614G, P681H

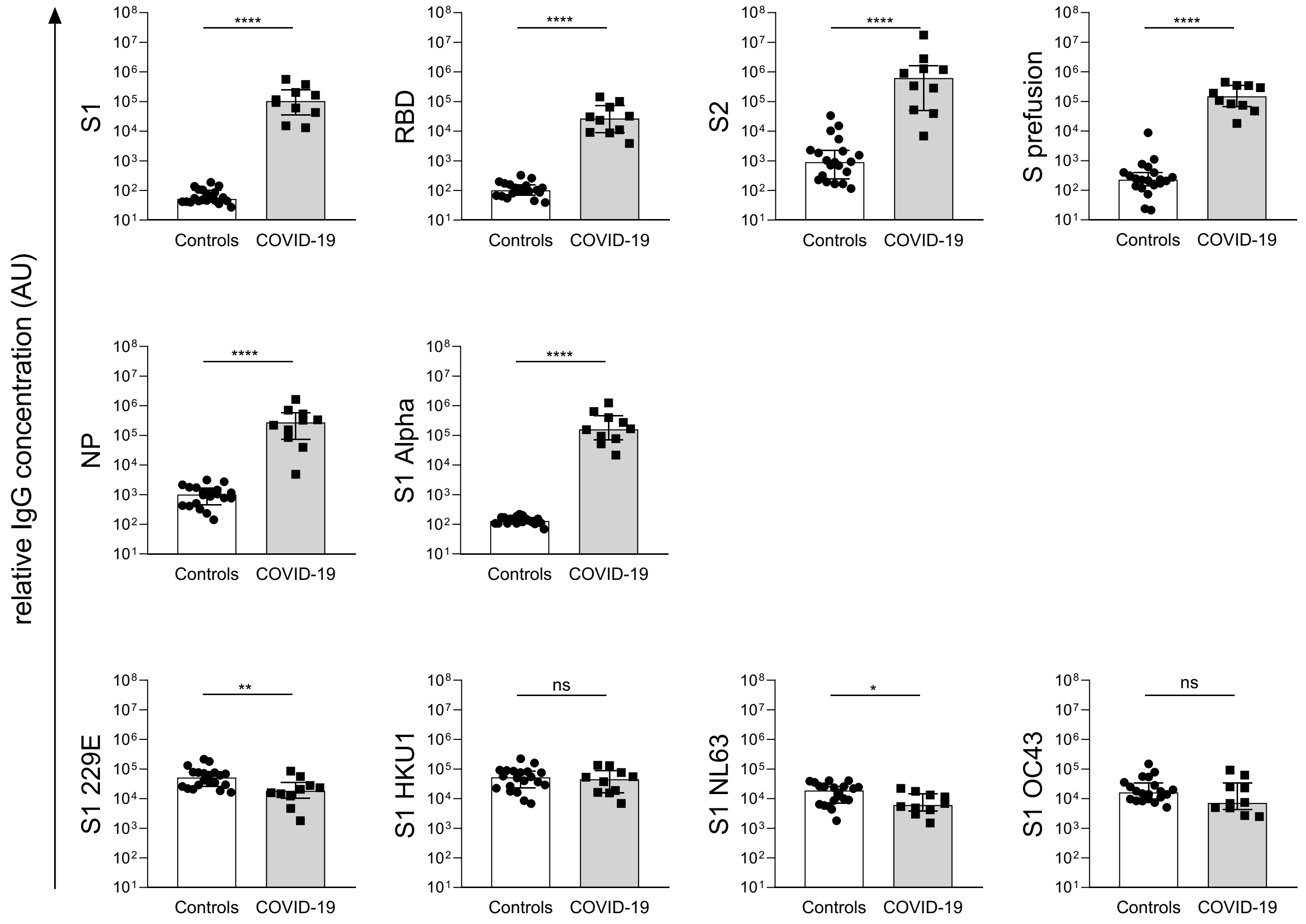

Supplementary Figure 2: Validation of a bead-based multiplexed immunoassay (Corona Array) for simultaneous quantification of antibody levels against antigens from SARS-CoV-2 and HCoVs. An in-house bead-based multiplexed immunoassay (Corona Array) was established to quantify IgG antibody levels against six SARS-CoV-2 antigens (S1 subunit, RBD domain, S2 subunit, S prefusion, NP from SARS-CoV-2 Wuhan strain, S1 from the SARS-CoV-2 Alpha variant) as well as S1 antigens from the four endemic human HCoVs (HCoV-229E, -HKU1, -NL63 and -OC43). The Corona Array was validated using 20 pre-COVID-19 sera from healthy individuals (controls) and 10 sera from convalescent hospitalized COVID-19 patients (COVID-19). Data are presented as median with IQR. The two groups were compared using a Mann-Whitney U test. *, $p \leq 0.05$; **, $p \leq 0.01$; ****, $p \leq 0.0001$.

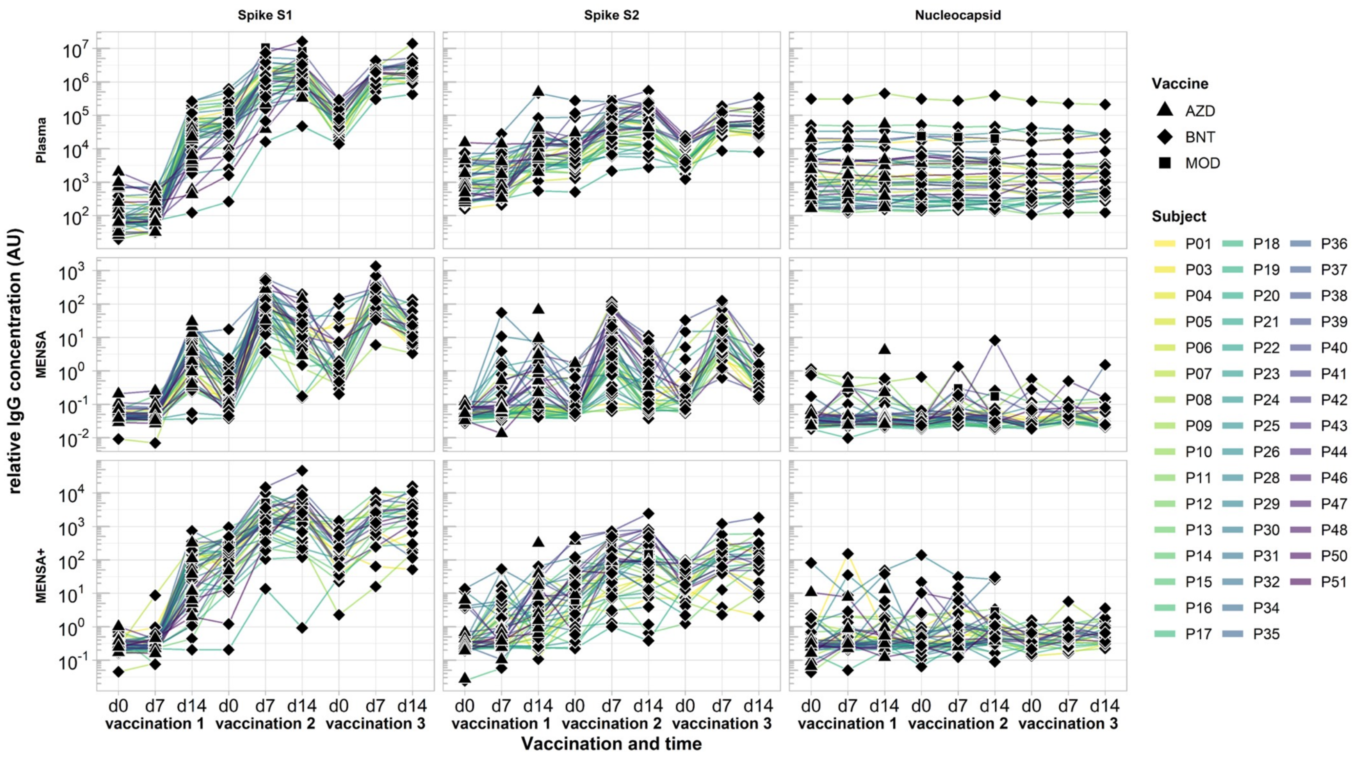

Supplementary Figure 3: Individual courses of plasma IgG, plasmablast, and memory B cell responses to SARS-CoV-2 Spike S1 and S2 subunits as well as NP after COVID-19 vaccination. Using a bead-based multiplexed immunoassay, IgG antibodies against the Spike S1 and S2 subunits as well as the nucleocapsid protein (NP) were quantified in plasma (top panel), MENSA (middle panel) and MENSA+ (bottom panel) samples obtained over the course of COVID-19 vaccination. PBMCs were cultured and either left untreated to assess spontaneous antibody secretion by plasmablasts (MENSA), or stimulated with IL-2 and the dual TLR7/8-ligand Resiquimod to assess antibody secretion by circulating Bmem cells (MENSA+). Vaccines are indicated in the legend to the right of the figure, together with the subject IDs. *Abbreviations:* AZD, ChAdOx1-S (Vaxzevria®; AstraZeneca); BNT, BNT162b2 (Comirnaty®; BioNTec/Pfizer); MOD, Spikevax® (Moderna).

Supplementary Table 3: Median ratios of IgG levels in plasma, MENSA, and MENSA+ samples for selected time points.

Analyte	Antigen	v1d7/v1d0	v1d14/v1d0	v2d7/v2d0	v2d14/v2d0	v2d14/v1d14	v3d7/v3d0	v3d14/v3d0	v3d0/v2d14	v2d14/v3d0	v2d14/v1d0	v3d14/v1d0	v3d0/v1d0
Plasma	S1	1.0	595.5	20.6	24.2	32.4	24.6	29.7	0.0	24.1	22520.0	39711.6	1180.2
	RBD	1.0	34.3	28.7	34.1	67.3	24.0	29.1	0.1	15.8	2487.2	7830.4	214.2
	S2	1.1	6.3	4.7	4.8	4.4	9.1	10.1	0.1	8.4	42.6	76.0	6.6
	S Prefusion	1.7	324.4	12.0	12.8	15.8	20.3	30.8	0.1	15.3	6602.8	15440.6	434.9
	NP	1.0	1.0	1.0	1.0	1.0	1.0	1.1	0.9	1.1	1.0	1.1	1.0
	S1 Alpha	1.0	479.4	21.0	23.7	32.1	25.4	32.8	0.0	20.6	26798.0	59495.8	1357.5
	S1 229E	1.0	0.9	1.0	0.9	1.0	1.0	1.0	0.9	1.2	0.9	0.8	0.8
	S1 HKU1	1.0	0.9	1.0	0.9	0.9	1.0	1.0	0.9	1.1	0.9	0.8	0.8
	S1 NL63	1.0	0.9	1.0	1.0	1.0	1.0	1.0	0.9	1.1	0.9	0.8	0.9
	S1 OC43	1.0	0.9	0.9	0.9	0.9	1.0	1.0	0.9	1.1	0.9	0.9	0.8
MENSA	S1	1.0	66.9	406.6	29.1	2.9	52.7	4.6	0.2	4.3	190.6	289.9	31.1
	RBD	1.0	2.5	199.0	10.1	5.9	66.1	5.1	0.3	3.8	20.9	40.3	5.4
	S2	1.2	3.3	29.9	1.8	1.3	26.2	2.6	1.0	1.0	4.2	12.9	2.7
	S Prefusion	1.1	118.1	219.6	15.3	2.4	57.3	5.0	0.3	3.8	151.2	346.6	40.0
	NP	1.0	1.1	1.3	1.1	1.0	1.4	1.1	0.7	1.4	1.1	1.0	0.8
	S1 Alpha	1.0	35.8	284.9	22.7	2.3	42.7	5.2	0.2	4.2	109.1	189.2	21.8
	S1 229E	1.0	1.1	1.0	1.0	1.0	0.9	1.1	1.9	0.5	1.3	2.4	1.9
	S1 HKU1	1.1	1.3	1.1	1.1	1.0	0.9	0.9	1.7	0.6	1.2	2.5	2.1
	S1 NL63	1.0	1.0	1.0	1.0	1.1	0.8	0.8	1.5	0.7	1.2	1.6	1.6
	S1 OC43	1.1	1.0	1.1	1.1	1.0	1.0	1.1	1.5	0.7	1.1	2.0	1.3
MENSA+	S1	1.0	166.6	13.1	9.9	27.3	7.3	10.0	0.1	15.1	5943.2	11837.8	719.9
	RBD	1.0	6.1	11.5	12.8	77.6	6.5	11.3	0.1	8.6	867.8	1748.0	125.8
	S2	1.3	5.4	8.2	9.4	21.5	3.9	4.2	0.2	4.8	195.2	360.2	52.6
	S Prefusion	1.2	239.7	10.9	9.5	20.6	5.1	6.3	0.2	5.5	6767.2	13237.8	1606.2
	NP	1.0	1.1	1.3	1.3	1.3	1.4	1.7	0.6	1.8	1.6	2.2	1.3
	S1 Alpha	1.0	111.2	12.6	7.4	33.3	8.0	11.0	0.1	10.5	3772.9	9482.5	634.7
	S1 229E	1.1	2.2	0.7	1.6	1.0	1.3	0.9	0.3	3.8	1.5	2.1	1.0
	S1 HKU1	1.0	1.1	0.9	1.3	0.7	0.9	0.4	0.9	1.2	1.4	0.9	1.0
	S1 NL63	0.8	1.3	1.1	1.2	1.1	0.9	1.3	0.7	1.5	1.5	1.2	1.0
	S1 OC43	1.0	1.1	0.7	0.5	1.1	0.6	1.1	0.8	1.3	1.3	1.0	1.0

Supplementary Table 4: Median antigen-specific IgG levels in plasma, MENSA, and MENSA+ samples.

Analyte	Antigen	v1d0	v1d7	v1d14	v2d0	v2d7	v2d14	v3d0	v3d7	v3d14
Plasma	S1	59.60	65.36	31803.05	50753.67	1295855.00	1340559.00	57354.90	1863487.00	2116206.50
	RBD	114.86	109.50	4320.52	9542.08	284628.50	328006.90	20765.28	668347.10	671934.65
	S2	976.42	1321.85	6237.12	7610.93	47281.71	42996.71	4449.76	50308.32	49031.64
	S Prefusion	157.57	288.04	58567.71	58305.28	1065980.00	1253871.00	63985.77	1581199.00	1793133.00
	NP	905.31	1047.86	955.69	1075.76	1162.65	1123.47	1006.14	1125.98	1419.12
	S1 Alpha	98.06	89.29	62329.50	87286.91	2306164.00	2445547.00	109180.80	3882965.50	3825640.50
	S1 229E	39172.96	42112.92	39900.20	37811.07	37717.59	35508.93	33794.86	33014.64	34317.86
	S1 HKU1	85364.02	87405.30	82088.92	86962.45	74633.30	79309.34	85412.56	80808.98	79084.50
	S1 NL63	13799.44	12229.25	13271.19	12959.35	12315.10	11574.01	11251.89	11887.88	11064.17
	S1 OC43	21723.08	21716.93	22745.18	20045.74	20218.87	20094.56	14769.14	13851.47	14764.14
MENSA	S1	0.04	0.05	3.77	0.25	84.65	7.91	1.61	126.36	14.22
	RBD	0.12	0.12	0.35	0.16	39.40	2.24	0.59	59.51	6.05
	S2	0.05	0.07	0.16	0.06	2.97	0.21	0.12	10.39	0.64
	S Prefusion	0.03	0.04	3.76	0.19	71.58	5.97	1.45	120.62	12.18
	NP	0.03	0.03	0.04	0.03	0.04	0.03	0.03	0.05	0.03
	S1 Alpha	0.12	0.12	4.34	0.49	152.03	13.41	2.97	234.74	29.67
	S1 229E	0.08	0.08	0.11	0.08	0.08	0.09	0.21	0.23	0.29
	S1 HKU1	0.22	0.25	0.29	0.24	0.27	0.31	0.69	0.70	0.79
	S1 NL63	0.08	0.08	0.09	0.09	0.09	0.09	0.15	0.13	0.15
	S1 OC43	0.10	0.12	0.10	0.10	0.10	0.11	0.13	0.19	0.20
MENSA+	S1	0.25	0.28	44.73	171.03	1496.54	1370.35	212.18	1862.73	2548.36
	RBD	0.65	0.71	3.53	51.01	530.13	586.65	92.30	757.90	937.05
	S2	0.28	0.48	1.75	8.42	64.12	89.36	18.01	113.97	125.77
	S Prefusion	0.19	0.22	58.12	135.50	1429.64	1321.58	348.33	2258.42	2748.18
	NP	0.27	0.32	0.36	0.33	0.41	0.53	0.37	0.51	0.59
	S1 Alpha	0.60	0.59	111.29	258.63	2345.08	2438.40	355.26	3336.86	4424.16
	S1 229E	3.08	16.85	10.58	4.78	6.04	22.63	2.90	3.92	2.90
	S1 HKU1	12.20	18.13	12.16	18.51	5.68	40.74	6.47	11.01	1.92
	S1 NL63	2.76	1.78	4.31	1.96	5.88	3.56	1.33	2.18	2.24
	S1 OC43	2.70	4.70	2.32	9.00	3.17	5.52	1.48	0.82	3.56

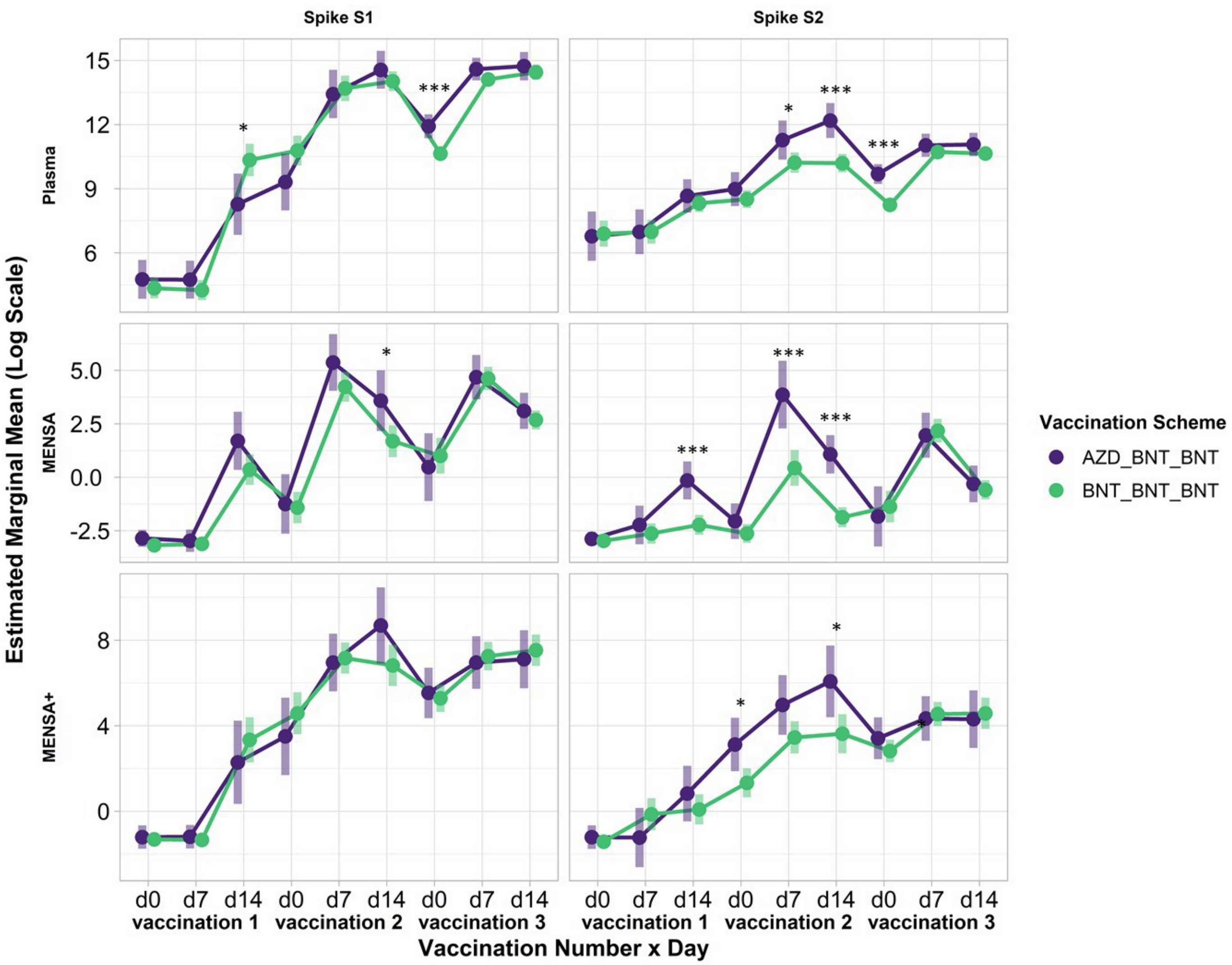

Supplementary Figure 4: Vaccination schemes impact on the early plasma IgG, plasmablast and Bmem responses to COVID-19 vaccination. The two largest subgroups received a homologous BNT/BNT/BNT (n = 32 (v1d0) – 19 (v3d14)) or heterologous AZD/BNT/BNT (n = 14 (v1d0) – 6 (v3d14)) vaccination scheme (see Fig. 1). IgG antibodies against the SARS-CoV-2 S1 and S2 subunit were quantified in serially diluted plasma (A), MENSA (B) and MENSA+ (C) using the Corona Array. Estimated marginal means of the anti-S1 and anti-S2 plasma IgG responses for the homologous BNT/BNT/BNT and the heterologous AZD/BNT/BNT subgroups are shown on a $\log_{10}$-scale. ANOVAs were conducted for each material and antigen. Pairwise post-hoc comparisons between both selected schemes for every measurement occasion were significant for plasma anti-S1 v1d14 (p = 0.0152) and v3d0 (p = 0.0004); anti-S2 v2d7 (p = 0.046), v2d14 (p = 0.0002) and v3d0 (p = 0.00001); for MENSA anti-S1 v2d14 (p = 0.023); anti-S2 v1d14 (p = 0.0003), v2d7 (p = 0.0007) and v2d14 (p = 0.000006); for MENSA+ anti-S2 v2d0 (p = 0.017) and v2d14 (p = 0.015). *, $p \leq 0.01$; ***, $p \leq 0.001$. Abbreviations: Bmem, memory B cells; MENSA, medium enriched for newly synthesized antibodies

A

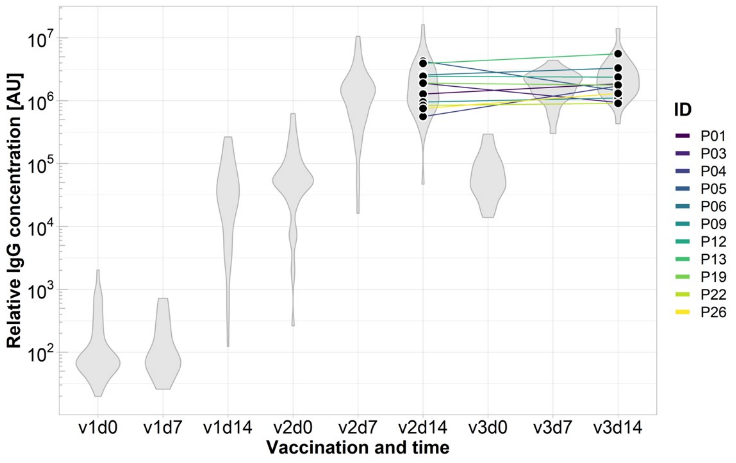

B

Variable	χ^2 / t	p
Sex	0.13	0.723
Age	1.91	0.085
Weight	0.21	0.842
BMI	-0.15	0.882

Supplementary Figure 5: Representativeness of the subcohort selected for the neutralization assay (n = 11). For cell-based neutralization assays a subcohort of n = 11 was selected from the overall AICOVI cohort (N = 46) meeting the following criteria: (1) homogenous vaccination scheme (BNT/BNT/BNT), (2) complete sample set (three vaccinations, each with d0, d7, and d14 samples), (3) no extreme values in the vaccine-induced immune response at any time point, (4) lack of other peculiarities (e.g. deviations in sampling). (A) The SARS-CoV-2 S1-specific IgG response of the overall AICOVI cohort on each day of as well as 7 and 14 days after (d0, d7, d14) each vaccination (v1–3) is depicted in violin plots with superimposed values of the selected 11 subjects. The selected 11 subjects exhibited representative plasma antibody levels against SARS-CoV-2 S1 compared to the overall cohort. (B) The representativeness of the subcohort for the overall study cohort was statistically ensured by a χ^2-test for sex distribution (df = 1) and by two-sided one-sample t-tests (df = 10) for deviations of the subset mean from the cohort means of age, weight and BMI. The subcohort did not differ significantly from the overall cohort with regard to sex distribution and the means of age, weight and BMI and is hence representative.

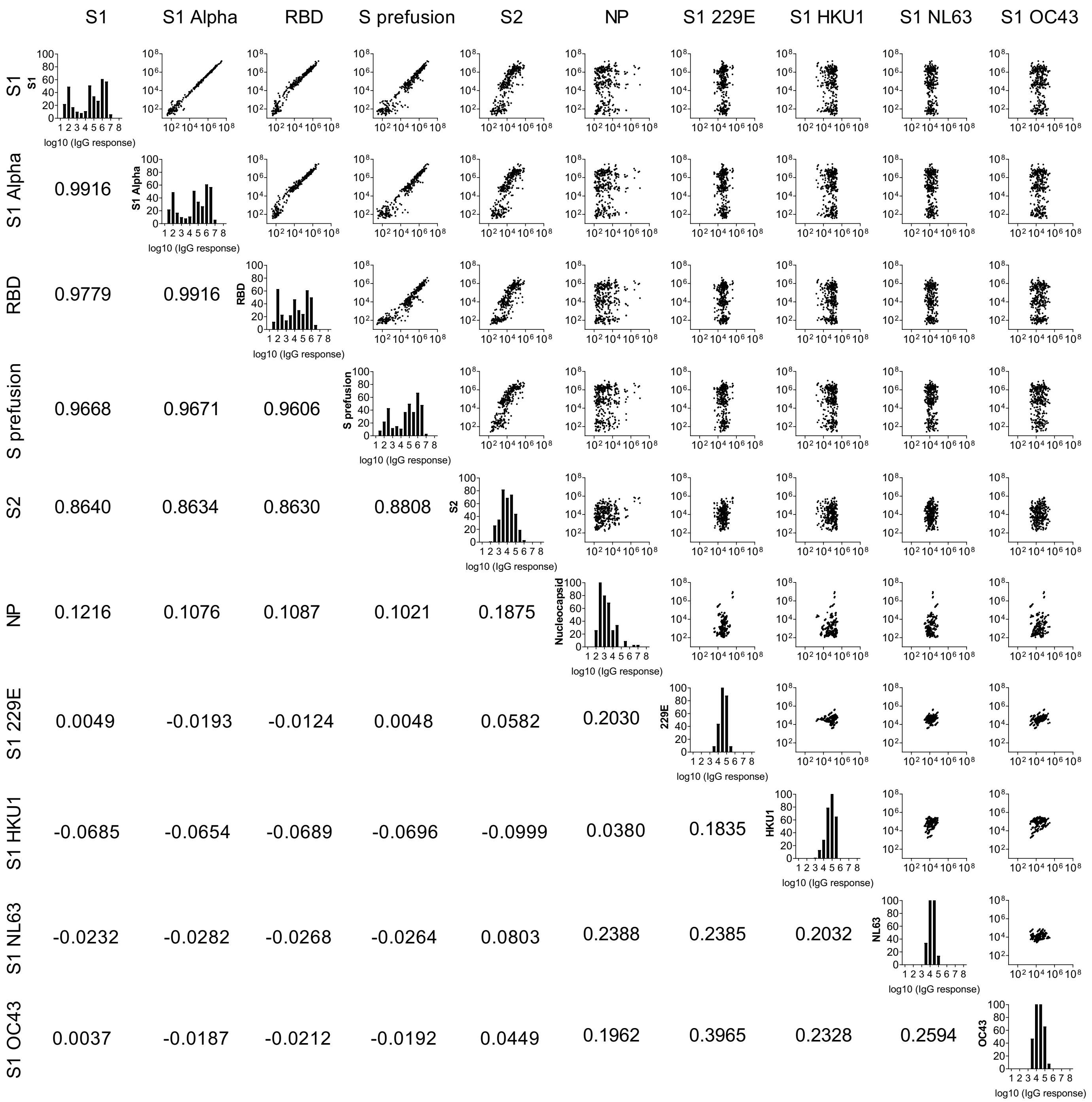

Supplementary Figure 6: Correlation matrix for plasma IgG binding to antigens from SARS-CoV-2 and endemic HCoVs. IgG antibody titers against 6 SARS-CoV-2 antigens (S1 subunit, S1-Alpha variant, RBD domain, Spike prefusion, S2 subunit, nucleocapsid), as well as S1 subunit from the four endemic HCoVs (HCoV-229E, -HKU1, -NL63 and -OC43) were quantified using a bead-based Corona Array. Graphs depict a collection of all data points from the AICOVI study (d0, d7, and d14 after the 1st, 2nd, and 3rd vaccination). Correlation matrices plotting all antigens against each other are depicted in the upper right part of the figure. The spearman correlation coefficient r is depicted on the mirrored side of the correlation matrix. For instance, the r value for the correlation of RBD with S1 is 0.9779. The diagonal represents histograms for each antigen based on the $\log_{10}$ relative IgG concentrations. The correlation matrices reflect the large epitope overlap between S prefusion, S1 and RBD (Spearman correlation coefficient r > 0.96). Moreover, they illustrate that there is virtually no correlation between antibody levels against the S1 domain from SARS-CoV-2 versus HCoVs (r ~ 0.0).

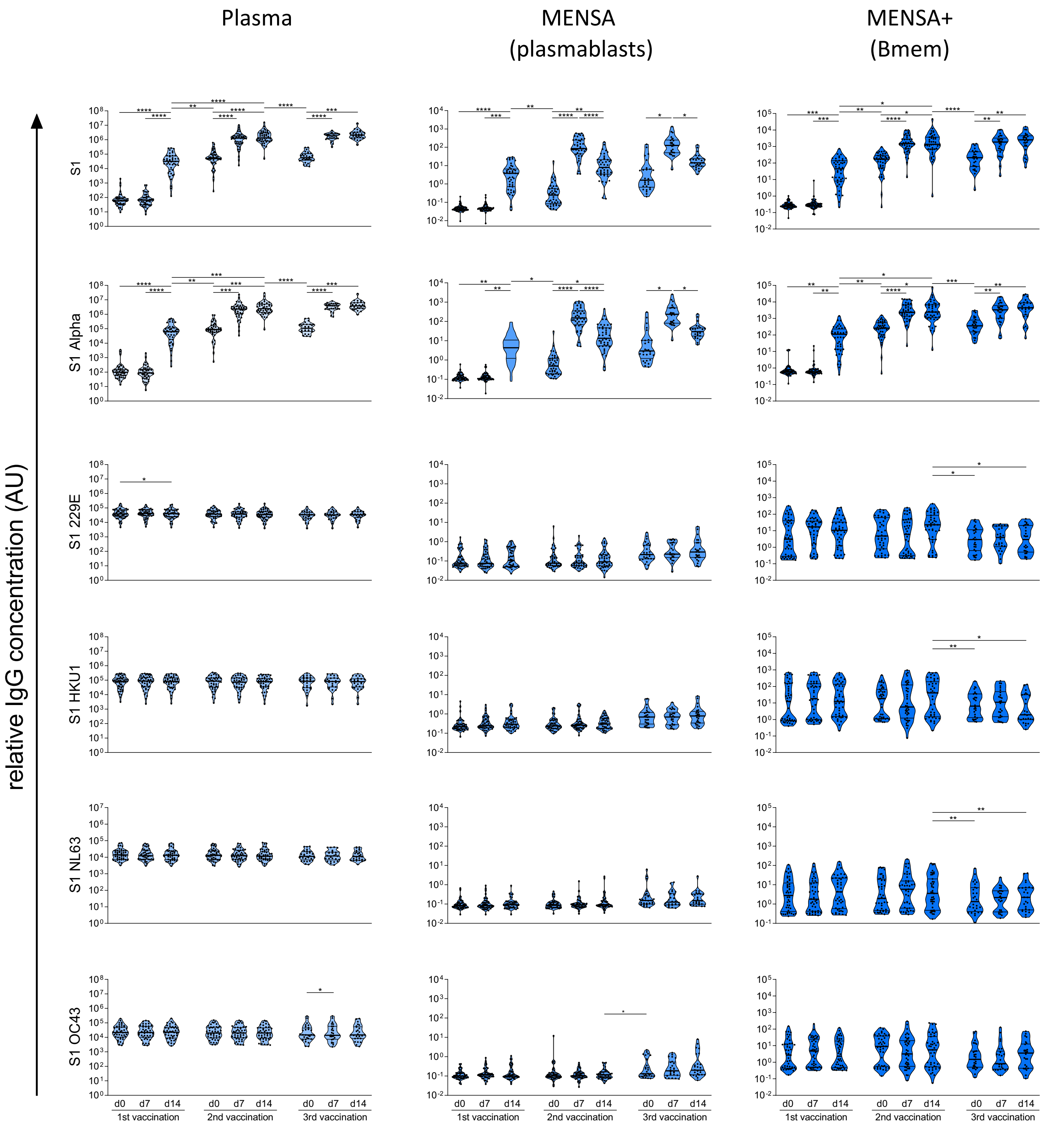

Supplementary Figure 7: COVID-19 vaccination does not affect plasma IgG, plasmablast and Bmem cell response against endemic HCoVs. Using a bead-based Corona Array, IgG antibodies against Spike S1 from the SARS-CoV-2 Wuhan strain, the Alpha variant, as well as the endemic HCoVs (HCoV-229E, -HKU1, -NL63 and -OC43) were quantified in plasma, MENSA and MENSA+ over the course of COVID-19 vaccination. Antibody profiles against the endemic HCoVs were unaffected by COVID-19 vaccination, suggesting that there is only little cross-reactivity on the B cell level for the S1 subunit. Violin plots with median and IQR. Statistics: Mixed-effects ANOVA with Geisser-Greenhouse correction for 13 *a priori* selected comparisons (as described in the methods section), followed by Sidak's multiple group comparisons. *, $p \leq 0.05$; **, $p \leq 0.01$; ***, $p \leq 0.001$; ****, $p \leq 0.0001$.

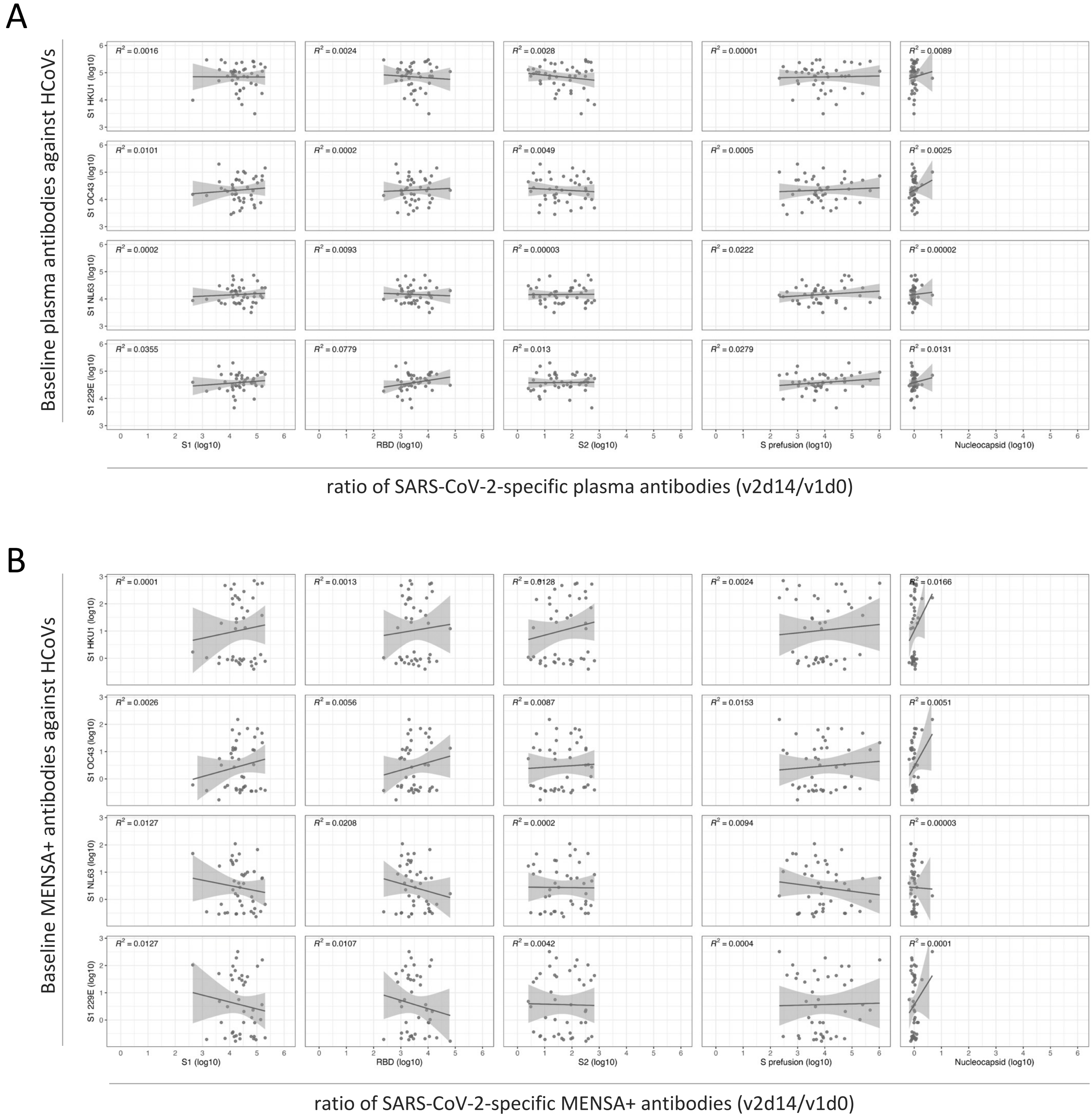

Supplementary Figure 8: B cell memory to HCoVs S1 does not inhibit the vaccination-induced response to novel SARS-CoV-2 antigens or epitopes. Scatterplot of baseline IgG levels (v1d0) for HCoV-229E, -HKU1, -NL63, and -OC43 S1 subunits and the ratio (v2d14/v1d0) of SARS-CoV-2 antibodies against S1, RBD, S2, and nucleocapsid in plasma (A) and MENSA+ samples (B). Lines with 95% confidence intervals (shaded gray areas) indicate a linear fit based on a simple linear regression model. R^2, square of the spearman correlation coefficient rho.
